# Synthetic Approaches for the Preparation of Phosphoramidate Prodrugs of 2′‐Deoxypseudoisocytidine

**DOI:** 10.1002/open.201700019

**Published:** 2017-05-05

**Authors:** Michaela Serpi, Roberto De Biasi, Fabrizio Pertusati, Magdalena Slusarczyk, Christopher McGuigan

**Affiliations:** ^1^ School of Pharmacy and Pharmaceutical SciencesCardiff UniversityKing Edward VII AvenueCardiffCF10 3NBUK), Fax: (+44) 02920874537; ^2^ Dipartimento di Scienze FarmaceuticheUniversità degli Studi di PerugiaVia del Liceo 106123PerugiaItaly

**Keywords:** 2′-deoxypseudoisocytidine, anticancer, *C*-nucleosides, phosphoramidates, prodrugs

## Abstract

A synthetic procedure for the preparation of phosphoramidate prodrugs of *C*‐nucleosides is reported. Different phosphorochloridates were reacted with 3′‐*O*‐protected *N*‐acetyl‐2′‐deoxypseudoisocytidine or 3′‐*O*‐protected 2′‐deoxypseudoisocytidine, followed by acidic hydrolysis of the protecting group. In the presence of the *N*‐acetyl moiety, the enolisable keto group of the nucleobase was able to react (like the 5′‐OH) with the phosphorochloridates to give bisphosphorylated derivatives. Epimerisation (β to α) occurred if the amino group of the nucleobase was unprotected. These side reactions demonstrate the peculiar behaviour of *C*‐nucleosides compared to their nucleoside analogues. It was demonstrated that the first enzymatic activation step for this new class of prodrugs can be mediated by carboxypeptidase and that it follows the same pathway and rate reported for ProTides of more conventional nucleoside analogues. These new phosphoramidate derivatives deserve further investigation for their therapeutic potential as anti‐cancer agents.

## 1 Introduction

**Figure 1:**
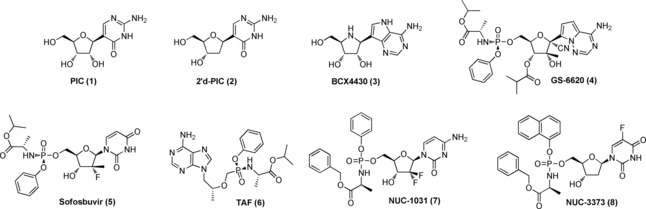
Structures of pseudoisocytidine (PIC, **1**), 2′‐deoxypseudoisocytidine (2′d‐PIC, **2**), BCX4430 (**3**), GS‐6620 (**4**), sofosbuvir (**5**), tenofovir alafenamide (TAF, **6**), NUC‐1031 (**7**), and NUC‐3373 (**8**).

The *C*‐nucleosides represent a group of nucleoside analogues in which the sugar moiety is linked to the nucleobase by a carbon–carbon bond.^1^ Several *C*‐nucleosides are naturally occurring compounds. Among them, pseudouridine was the first to be isolated from yeast tRNA in 1957.^2^ Subsequently, other *C*‐nucleosides, including oxazinomycin,^3^ pyrazomycin,^3^ showdomycin,^4^ and formycin A,^5^ were isolated from culture filtrates of different bacterial strains. These compounds are antibiotics and exhibit anti‐cancer and/or antiviral activity. Their advantageous properties arise from the presence of a C−C glycosidic bond, which gives a greater resistance than *N*‐nucleosides towards chemical hydrolysis and enzymatic hydrolysis by phosphorylase and deaminase enzymes. On the basis of these interesting chemical and biological properties, a wide variety of synthetic analogues have been prepared thanks to the large array of novel synthetic methodologies developed in the last two decades. Several of these compounds have found numerous applications in medicinal chemistry and chemical biology.^1^ Among them, pseudoisocytidine (PIC, **1**), a nucleoside isostere of cytidine was developed as a candidate for anti‐leukaemic therapy^6^ (Figure 1). PIC was shown to be incorporated into both RNA and DNA and this incorporation was considered to be responsible for its therapeutic activity, which has been observed against several mouse leukaemias in vitro and in vivo.^7^, ^8^ In addition, PIC was found to disrupt DNA methylation by inhibition of the enzyme DNA methyltransferase, most probably due to the presence of a nitrogen atom in the 5‐position of the base.^9^ However, the development of PIC was halted due to hepatotoxicity observed during phase I clinical evaluation.^10^ The efficiency with which PIC is incorporated into RNA, and the rapid RNA turnover, associated with protein synthesis in the liver, were considered the main causes of its hepatotoxicity. This finding prompted the investigation of 2′‐deoxypseudoisocytidine (2′d‐PIC, **2**),^11^ which, in preliminary tissue culture experiments, was found to exhibit inhibitory activity against P815 cell lines.11a PIC, 2′d‐PIC and their analogues were also used as novel base‐pairing agents in oligonucleotides to investigate DNA and RNA structures and functions.^12^ Although several *C*‐nucleoside analogues have been described as anti‐cancer and/or antiviral agents, none have ever been developed as anti‐cancer or antiviral drugs. The recent advent of two novel *C*‐nucleosides, BCX4430 (**3**)^13^ and GS‐6620 (**4**),^14^ as potential therapeutic agents for the treatment of the Ebola virus and hepatitis C virus (HCV) infections, respectively, has stimulated renewed interest in this class of compounds (Figure 1).

As part of our current research we were interested to further investigate the potential utility of 2′d‐PIC (**2**) as an anti‐leukaemic agent by preparing a series of phosphoramidate prodrugs for biological evaluation as anti‐cancer agents. “ProTides” in the clinic have consistently showed greater efficacy and more favourable safety profiles relative to the corresponding standard‐of‐care nucleoside analogues. Several pharmaceutical companies have already validated the phosphoramidate approach for antiviral applications. In 2014, Gilead launched on the market its anti‐HCV ProTide, sofosbuvir (**5**)^15^ and in the following year tenofovir alafenamide (TAF, **6**),^16^ an anti‐HIV ProTide (Figure 1). NuCana introduced the ProTide technology to clinical oncology with NUC‐1031 (**7**),^17^ a ProTide of gemcitabine, and NUC‐3373 (**8**),^18^ a ProTide of 2′‐deoxy‐5‐fluorouridine, currently in phase III and phase I clinical studies, respectively, for patients with advanced solid tumours (Figure 1).

## 2 Results and Discussion

### 2.1 Synthesis of 2′‐Deoxypseudoisocytidine (2)

**Scheme 1:**
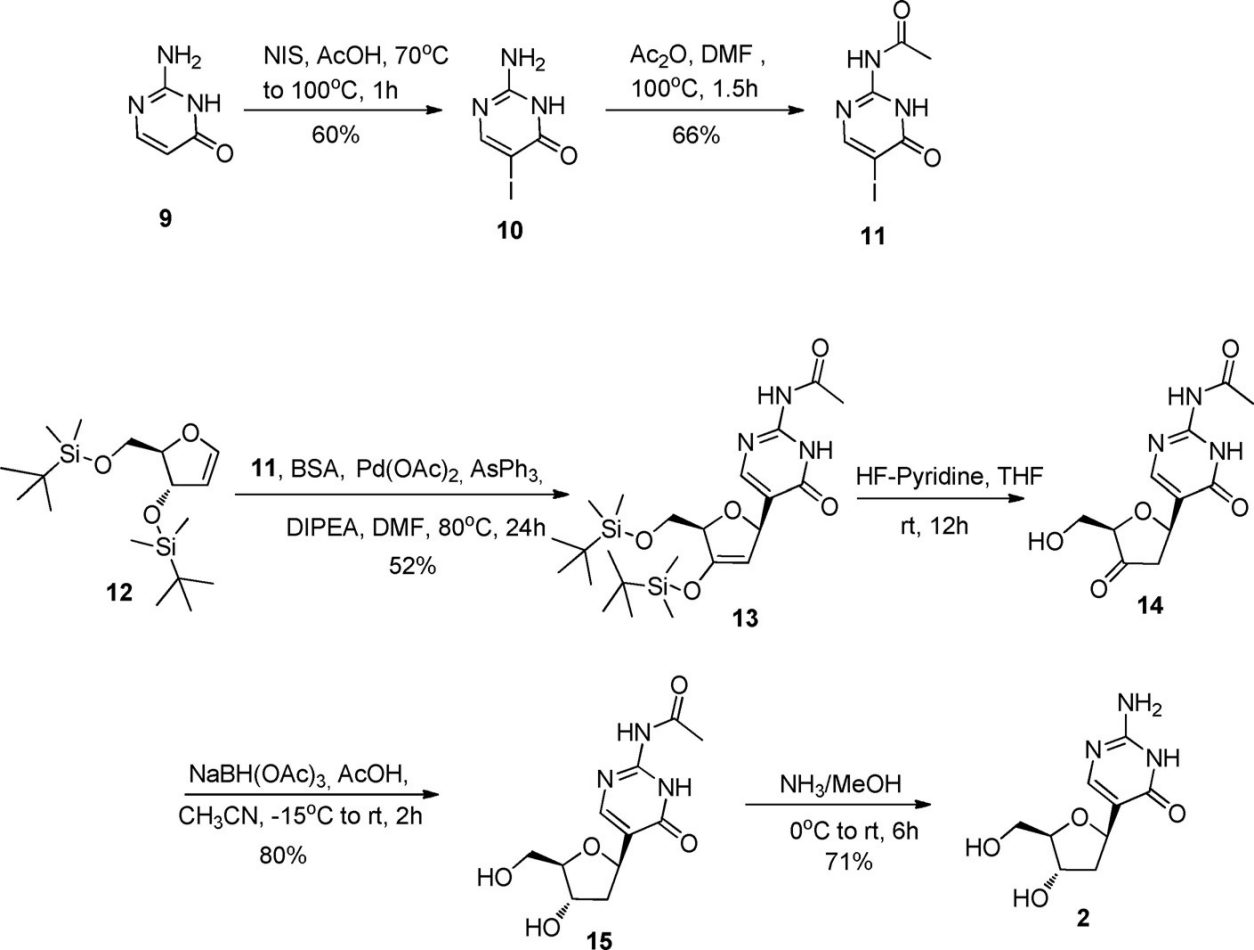
Synthesis of 2′d**‐**PIC (**2**). NIS, *N*‐iodosuccinimide; BSA, Bis(trimethylsilyl)acetamide ; DIPEA, *N*,*N*‐diisopropylethylamine.

Several approaches have been developed for the preparation of *C*‐glycosides^19^ and *C*‐nucleosides.^20^ Among them, for the synthesis of 2′d‐PIC (**2**), we selected the methodology developed by Daves et al., which utilises a Pd‐catalysed Heck‐type coupling of aryl halides to cyclic enol ethers, either pyranoid or furanoid glycals.^21^ As outlined in Scheme 1, the protected furanoid glycal **12** and the halogenated *N*‐acetyl pseudoisocytosine **11**, served as starting materials for the Heck reaction. 2‐*N*‐Acetyl‐5‐iodoisocytosine (**11**) was synthesised in good yield in two steps from commercially available isocytosine (**9**), which was first iodinated with *N*‐iodosuccinimide in acetic acid to afford the intermediate compound **10**. Subsequent acetylation of the exocyclic amino function of **10** using acetic anhydride yielded the desired nucleobase **11**.^22^ Compound **12** was prepared from 3′,5′‐bis‐*O*‐(*tert*‐butyldimethylsilyl)thymidine^23^ by using typical silylation conditions first reported by Pedersen et al.^24^ and then applied by Hammer et al.^25^ for the preparation of furanose glycals with a wide range of *O*‐silyl protections.

The coupling reaction of 5‐iodo base **11** with the protected ribofuranosyl glycal **12** using Pd(OAc)_2_ as a catalyst, AsPh_3_ as a soft ligand and *N*,*N*‐diisopropylethylamine as a base, formed selectively the β‐*C*‐nucleoside **13**. After removal of the silyl groups with fluoride ions, the resulting 2′‐deoxy‐3′‐keto *C*‐nucleoside **14** was treated with sodium triacetoxyborohydride to reduce diastereoselectively the 3′‐keto group from the β‐face of the furanosyl ring, forming *N*‐acetyl‐2′‐deoxypseudoisocytidine **15**.^22^ The cleavage of the acetyl group to afford nucleoside **2** was then accomplished by basic hydrolysis using NH_3_ in MeOH. The assignment of the configuration at the 1′‐position of **2** was based on the comparison of its ^1^H NMR spectrum with that reported in the literature.^22^

### 2.2 Synthesis of *N*‐Acetyl‐2′‐deoxypseudoisocytidine Phosphoramidates

**Scheme 2:**
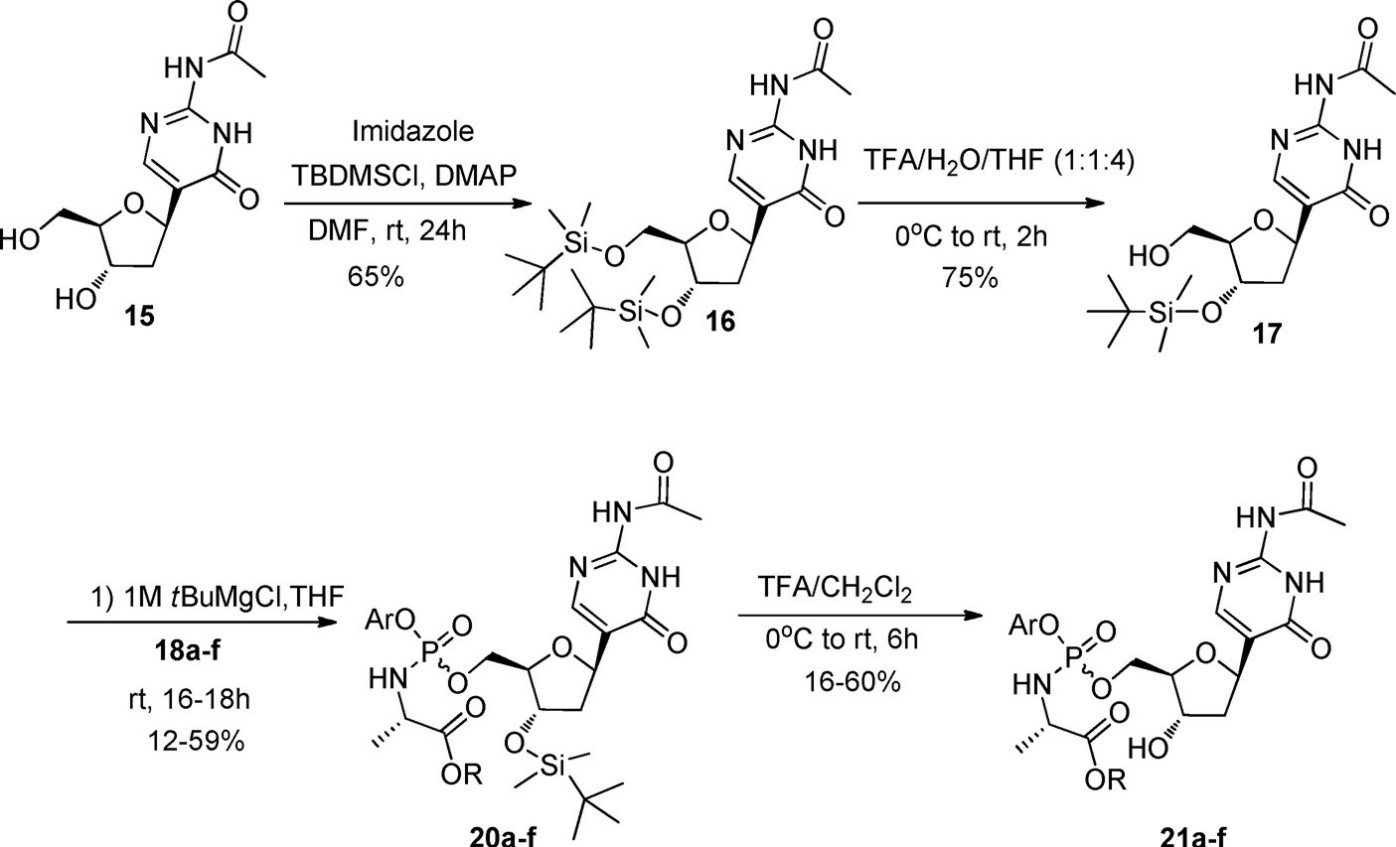
Synthesis of ProTides **21 a**–**f**. TBDMSCl, *tert*‐butyldimethylsilyl chloride; DMAP, 4‐dimethylaminopyridine; TFA, trifluoroacetic acid.

The two synthetic strategies commonly used for the preparation of phosphoramidate prodrugs (phosphorochloridate in the presence of either *tert*‐butylmagnesium chloride or *N*‐methylimidazole as a base)^26^ failed when applied to **2**, probably due to the low solubility of the starting material in the reaction medium, returning only unreacted starting materials. Attempts to improve the solubility of **2** using different solvents were unsuccessful. Application of the ProTide approach to precursors **14** and **15** also failed, indicating that development of a suitable synthetic strategy to afford phosphoramidates of **2** was more challenging than originally expected. These results prompted us to use a different synthetic methodology with compound **17** as the key intermediate (Scheme 2). We envisaged that introduction of a *tert*‐butyldimethylsilyl ether at the 3′‐OH group in **15** would help to improve its solubility and to achieve exclusive phosphorylation at the 5′‐position.

**Figure 2:**
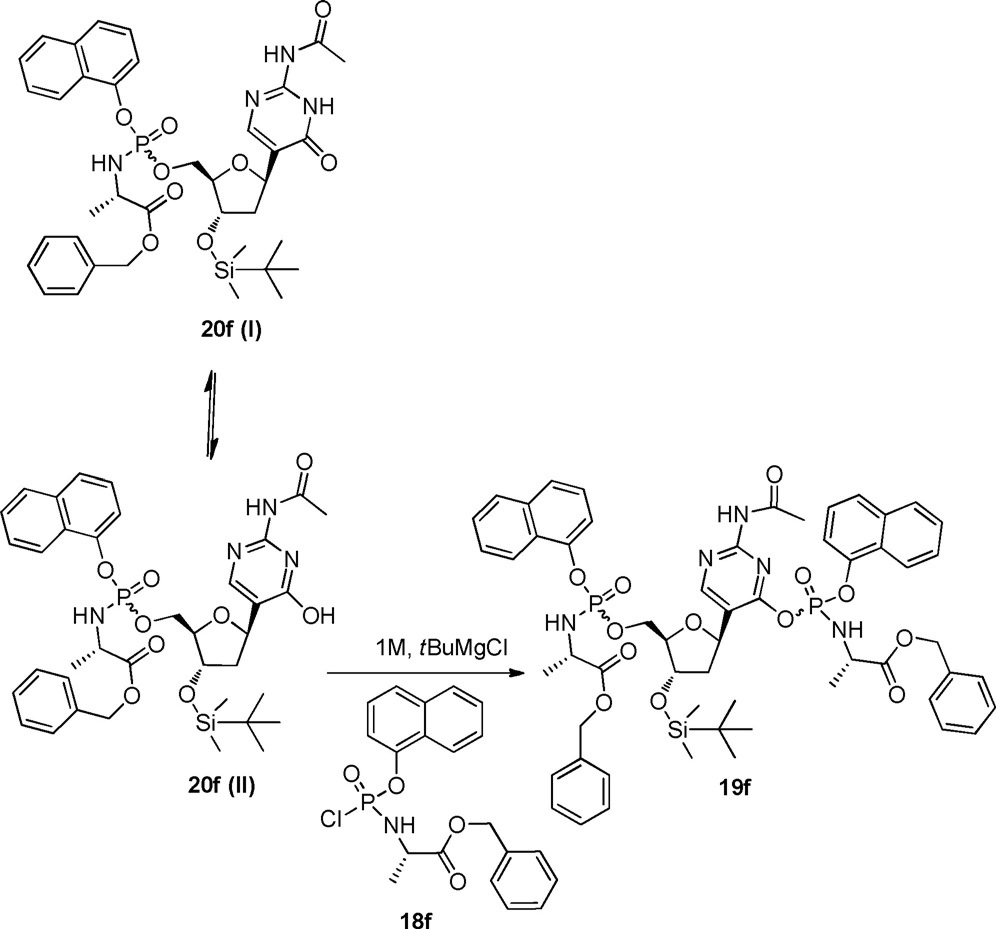
Proposed rationale for the *O*‐phosphorylation side reaction.

In order to prepare compound **17**, the two hydroxy groups of deoxyribose present in *N*‐acetyl‐2′‐deoxypseudoisocytidine (**15**) were first protected with a *tert*‐butyldimethylsilyl group using *tert*‐butyldimethylsilyl chloride in DMF for 24 h at room temperature in the presence of 4‐dimethylaminopyridine (DMAP) to provide, after flash chromatography, compound **16** in reasonable yield. Then, selective silyl group deprotection was achieved with aqueous trifluoroacetic acid to give, after isolation by silica gel chromatography, **17** with a free primary hydroxy group in moderate yield. Next, phosphorochloridates **18 a**–**f**, prepared as a mixture of *R*_P_ and *S*_P_ diastereoisomers according to a literature procedure,^26^ were reacted with **17** in the presence of *tert*‐butylmagnesium chloride (1.0 m in THF), yielding 3′‐*O*‐*tert*‐butyldimethylsilyl phosphoramidates **20 a**–**f** (Scheme 2) as diastereoisomeric mixtures after column chromatography, except for **20 d**, which was isolated after purification as a single diastereoisomer. Despite the almost complete consumption of the starting material, the desired products **20 a**–**f** were recovered in low yields, which was ascribed in each case to the formation of a bisphosphorylated by‐product, as exemplified in Figure 2. The bisphosphorylated compound **19 f** was isolated and its structure was characterised by mass spectrometry and ^31^P and ^1^H NMR analysis,^31^ which clearly suggested that the phosphorylation involved the oxygen atom of the pyrimidine ring rather than either one of the nitrogen atoms. *N*‐Acetylisocytidine possesses an enolisable keto group which, like the 5′‐OH group, is able to react with a phosphorochloridate to give an *O*‐phosphorylated derivative. In support of this result, we found in the literature that the reaction of 2‐acetylamino‐4‐hydroxypyrimidines with phosphorochloridates gives *O*‐phosphoryl rather than *N*‐phosphoryl derivatives.^27^ The substantial steric requirement of the phosphoryl chloride and the steric hindrance exerted to some extent by the acetyl group were considered to be the key features for preventing phosphorylation at either one of the ring nitrogen atoms.^27^

Acidic deprotection of **20 a**–**f** afforded after preparative HPLC purification compounds **21 a**–**f** in moderate yields (Scheme 2 and Table 1). Attempts to remove the acetyl protection from **21 a** with Schwartz's reagent as described by Ferrari et al.,^28^ failed due to the ring opening of the base. The difficulties encountered in removing the *N*‐acetyl group from **21 a**–**f** using mild conditions, and the fact that the labile P−O bond of the ProTide would not tolerate other harsh de‐acetylating agents such as methanolic ammonia, prompted us to abandon our attempts toward modification of **21 a**–**f**. We therefore continued our effort to conceive a more efficient route that would allow the preparation of the *N*‐deacetylated analogues.

**Table 1:** Reaction outcomes for the synthesis of precursors **20 a**–**f** and ProTides **21 a**–f.

Cmpd	Ar	R	Yield [%]	d.r.	Cmpd	Yield [%]	d.r.
**20 a**	Ph	CH_2_Ph	25	1.5:1	**21 a**	50	2.3:1
**20 b**	Ph	(CH_2_)_5_CH_3_	21	1.5:1	**21 b**	60	1.5:1
**20 c**	Ph	(CH_2_)_4_CH_3_	28	1:1	**21 c**	16	1:1
**20 d**	Naph	CH(CH_3_)_2_	12	1:0	**21 d**	37	1:0
**20 e**	Naph	CH_2_C(CH_3_)_3_	13	1:1	**21 e**	46	2.3:1
**20 f**	Naph	CH_2_Ph	59	1.5:1	**21 f**	25	4:1

### 2.3 Synthesis of 2′‐Deoxypseudoisocytidine Phosphoramidates

**Scheme 3:**
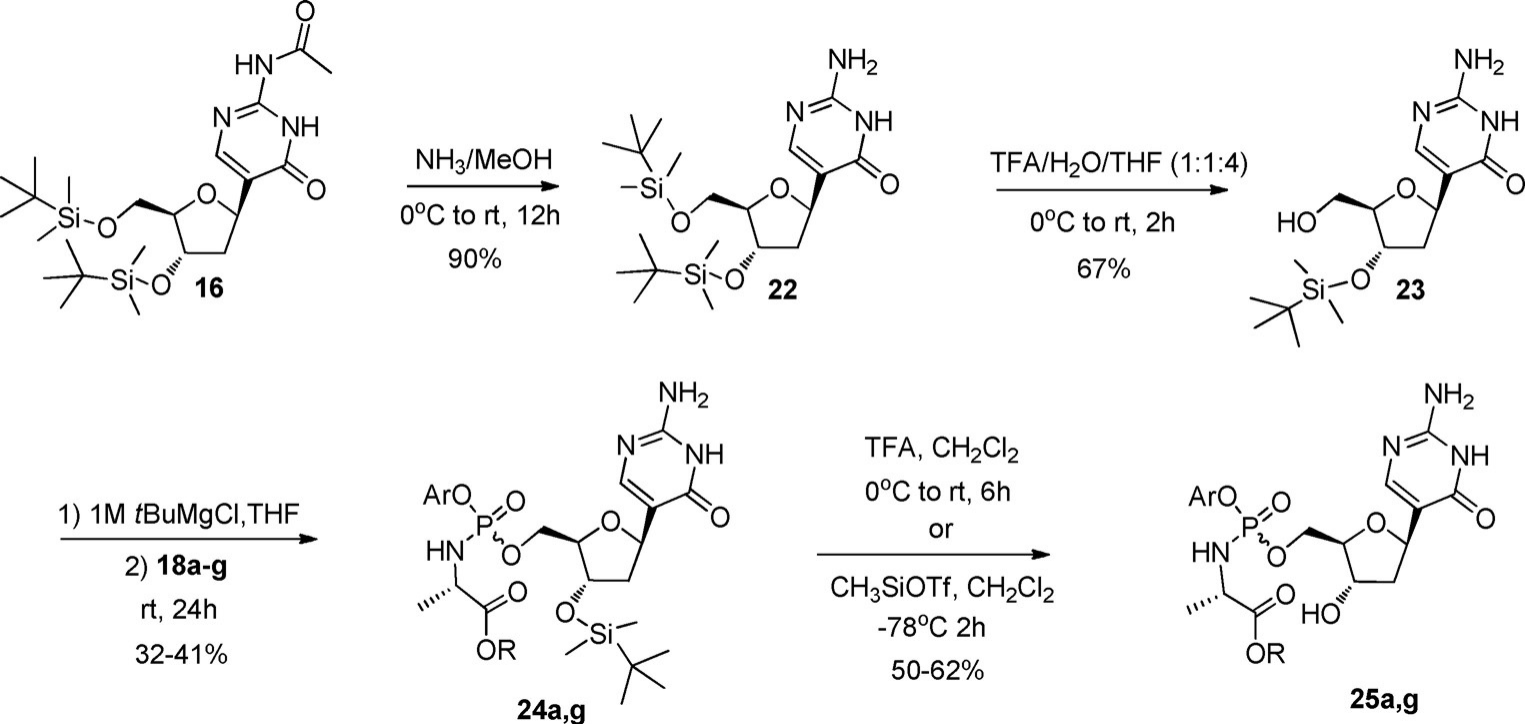
Synthesis of ProTides **25 a** and **25 g**.

As shown in Scheme 3, compound **22**, obtained by treatment of **16** with methanolic ammonia, underwent selective 5′‐desilylation using aqueous trifluoroacetic acid in THF to afford the monosilyl compound **23** in excellent yield. Next, phosphorochloridates **18 a** and **18 g** were reacted with **23** in the presence of *tert*‐butylmagnesium chloride (1.0 m in THF) to yield, after column chromatography, the 3′‐*O*‐*tert*‐butyldimethylsilyl‐protected phosphoramidates **24 a** and **24 g** in moderate yield as diastereoisomeric mixtures (Table 2). No traces of bisphosphorylated products either due to *O*‐ or *N*‐phosphorylation were observed.

**Table 2:** Reaction outcomes for the synthesis of **24 a** and **24 g** and ProTides **25 a** and **25 g** after HPLC purification.

Cmpd	Ar	R	d.r.	Yield [%]	Cmpd	Yield [%]	d.r.
**24 a**	Ph	CH_2_Ph	2.3:1	41	**25 a**	62	1:0
**24 g**	Ph	CH(CH_3_)_2_	1:3	32	**25 g**	50	4:1

**Scheme 4:**
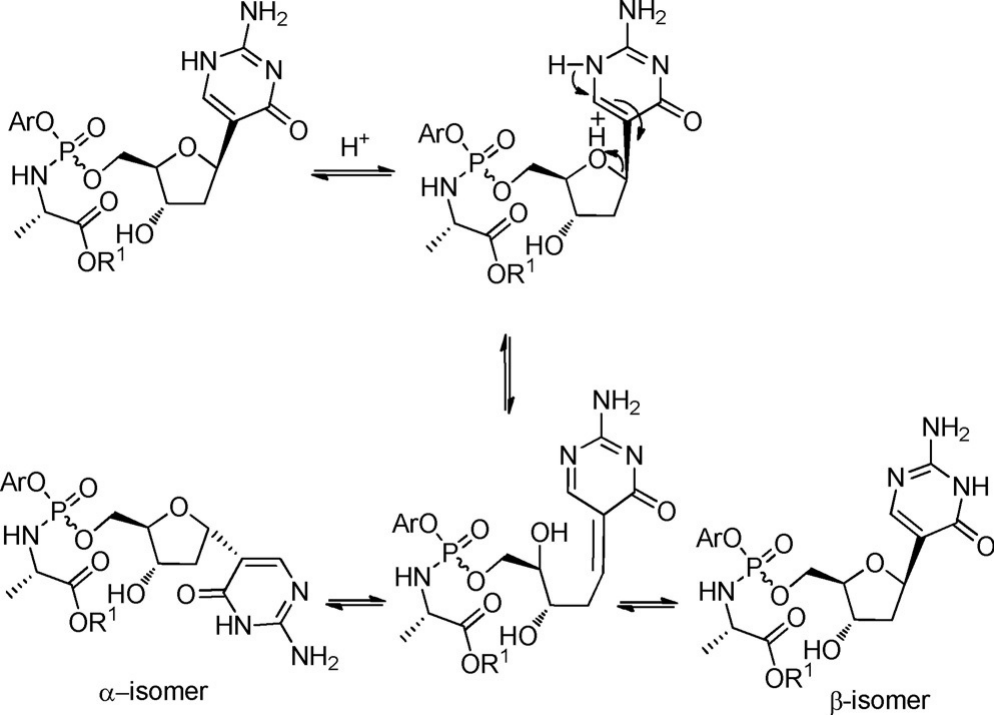
Proposed mechanism for the epimerisation of *C*‐nucleoside phosphoramidates under acidic conditions.

Acidic deprotection of the *tert*‐butyldimethylsilyl moieties in **24 a** and **24 g** with trifluoroacetic acid in dichloromethane (1:2 v/v; room temperature, overnight), afforded the final compounds **25 a** and **25 g** as mixtures of α and β isomers in a 3:1 ratio after column chromatography. The β‐isomers of **25 a** and **25 g** were isolated in low yield after preparative HPLC purification (**25 a** as a single diastereoisomer and **25 g** as a mixture; Scheme 3 and Table 2). Most probably, the presence of a dissociable proton on N‐1 facilitates the α,β‐epimerisation in acidic conditions through a ring opening–closure of the carbohydrate ring (Scheme 4) as previously reported for other *C*‐nucleosides.11b, 11c, 29

If a mild procedure for the cleavage of *tert*‐butyldimethylsilyl ethers to alcohols (based on an exchange reaction with trimethylsilyl triflate at −78 °C)^30^ was used, no epimerisation was observed.

### 2.4 Enzymatic Studies on the Activation of *C*‐Nucleoside ProTides

To exert their biological activity, ProTides must be metabolised in vivo into the monophosphate form, which in turn generates the active triphosphate form by two consecutive phosphorylation reactions.^31^ In the process of intracellular activation of ProTides, the first step is catalysed by a carboxyesterase‐type enzyme, such as cathepsin A, which was shown to be responsible for the cleavage of the amino acid ester moiety.^32^ In order to demonstrate that the ProTides of *C*‐nucleosides are activated in a similar manner, the interaction of compound **21 e** with a carboxyesterase‐type enzyme was investigated. Carboxypeptidase Y was used as a surrogate of cathepsin A because it belongs to the same family of C‐type carboxypeptidases and it was reported to share similarities in the active site.^33^

**Figure 3:**
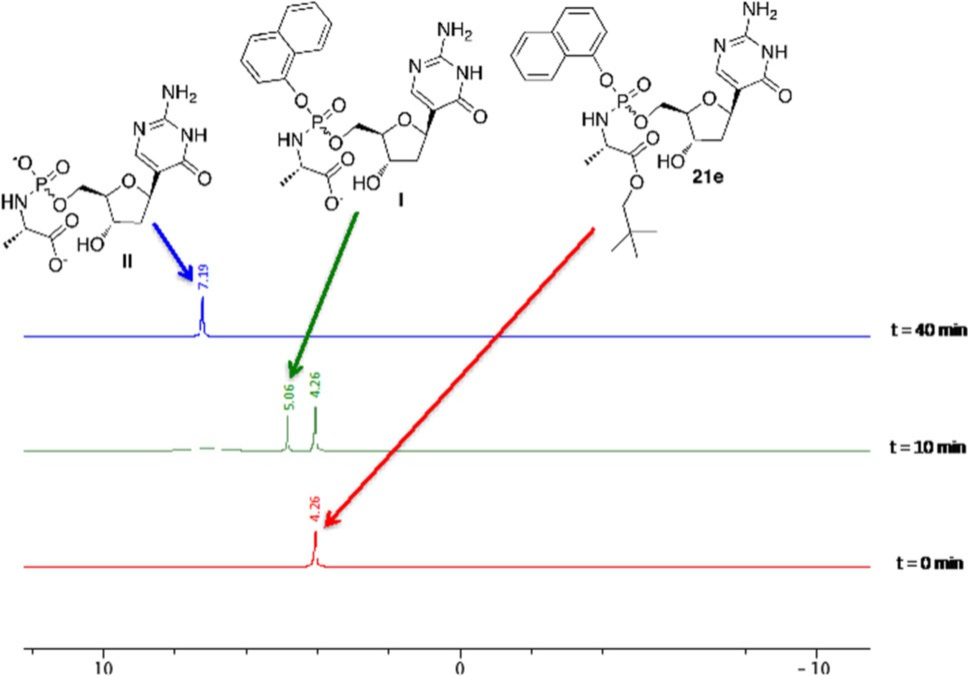
Deconvoluted ^31^P NMR spectra (202 MHz, [D_6_]acetone/pH 7.6 Trizma buffer) to show the carboxypeptidase‐mediated activation of compound **21 e**.

Compound **21 e** in [D_6_]acetone was therefore incubated in an NMR tube with carboxypeptidase Y in Trizma buffer (pH 7.6), and the progress of the reaction was monitored by ^31^P NMR analysis over 14 h. The stacked spectra (Figure 3) show the formation of a new peak after 10 min of incubation, which corresponds to intermediate **I** (*δ*_P_=5.06 ppm, *t*=10 min). Complete conversion of the ProTide **21 e** (which in [D_6_]acetone appears as a single peak at *δ*_P_=4.26 ppm) into the corresponding aminoacyl phosphoramidate ester (**II**: *δ*_P_=7.19 ppm) was observed in 40 min. In vivo, the aminoacyl phosphoramidate ester metabolite is then believed to undergo P−N bond cleavage, mediated by a phosphoramidase‐type enzyme to eventually release the parent drug in its monophosphate form.

## 3 Conclusions

An alternative route to *C*‐nucleoside ProTides has been developed and used to prepare phosphoramidate derivatives of 2′d‐PIC (**2**) and *N*‐acetyl 2′d‐PIC (**15**). Unexpected side reactions such as phosphorylation of the enolisable keto group of the nucleobase and epimerisation through ring opening highlighted the different reactivity of *C*‐nucleosides compared to nucleoside analogues. The first carboxypeptidase‐mediated bioactivation step for this new class of prodrugs followed the same pathway and rate as reported for ProTides of conventional nucleoside analogues. Biological evaluation of these novel nucleoside analogues should enhance our understanding of the potential of *C*‐nucleosides as anti‐tumour agents and in particular of 2′d‐PIC as an anti‐leukaemic drug. Together with derivatives **25 a** and **25 g**, we plan to evaluate the *N*‐acetylated derivatives **21 a**–**f** for their anti‐tumour activity. We considered that the acetyl moiety would further enhance the lipophilicity of these compounds and remove the potential for their protonation in vitro, whereas in vivo the acetyl moiety would most probably be able to undergo cleavage (thus acting as a dual prodrug). The results of these investigations will be disclosed in due course.

## Experimental Section

### Chemistry

All anhydrous solvents were purchased from Sigma–Aldrich and amino acid esters from Novabiochem. All commercially available reagents were used without further purification.

Precoated aluminium‐backed plates (60 F_254_, 0.2 mm thickness, Merck) were used for thin‐layer chromatography (TLC) and were visualised under both short‐ and long‐wavelength UV light (254 and 366 nm, respectively). Flash column chromatography was performed using silica gel supplied by Fisher (60 A, 35–70 μm). Analytical HPLC analysis was performed using either a ThermoScientific or a Varian Prostar system. ^1^H (500 MHz), ^13^C (125 MHz), and ^31^P NMR (202 MHz) spectra were recorded on a Bruker Avance 500 MHz spectrometer at 25 °C. Chemical shifts (*δ*) are quoted in parts per million (ppm) relative to internal references CD_3_OD (*δ=*3.34 ppm, ^1^H NMR; *δ=*49.86 ppm, ^13^C NMR) and CDCl_3_ (*δ=*7.26 ppm, ^1^H NMR; *δ=*77.4 ppm, ^13^C NMR), or external 85 % H_3_PO_4_ (*δ=*0.00 ppm, ^31^P NMR). Coupling constants (*J*) are expressed in Hertz. The following abbreviations are used in the assignment of NMR signals: s (singlet), d (doublet), t (triplet), q (quartet), m (multiplet), br s (broad singlet), dd (doublet of doublet), dt (doublet of triplet). Low‐resolution mass spectrometry was performed on a Bruker Daltonics micrOTOF–LC system.

For practical purposes, in some cases standard procedures are given. Procedures that differ from the standard are fully described.

#### *N*‐(6‐Oxo‐1,6‐dihydropyrimidin‐2‐yl)acetamide (10)

*N*‐Iodosuccinimide (22.0 g, 98 mmol) was added to a solution of 2‐aminopyrimidin‐4(3*H*)‐one (**9**, 10.0 g, 90 mmol) in acetic acid (170 mL) at 70 °C. The resulting suspension was heated at 100 °C and stirred for 1 h. The reaction mixture was cooled to room temperature and H_2_O (500 mL) was added. The solid was filtered, washed with H_2_O and dried with a heat gun at 50 °C for 12 h to afford compound **10** as a light pink solid (12.8 g, 60 % yield). ^1^H NMR (500 MHz, DMSO): *δ*=11.24 (br s; N*H*), 7.93 (s, 1 H; *H*‐6), 6.69 ppm (s, 2 H; N*H*_2_).

#### *N*‐(5‐Iodo‐6‐oxo‐1,6‐dihydropyrimidin‐2‐yl)acetamide (11)

A suspension of **10** (11.8 g, 49.8 mmol) and acetic anhydride (11.8 mL, 124.5 mmol) in DMF (200 mL) was heated to 100 °C. After 1.5 h the clear solution was evaporated to dryness under high vacuum and the residue was suspended in EtOH. The solid was filtered off and washed with Et_2_O to afford **11** as a white solid (9.2 g, 66 % yield). ^1^H NMR (500 MHz, DMSO): *δ*=12.15 (s, 1 H; N*H*), 11.74 (br s; N*H*), 8.31 (s, 1 H; *H*‐6), 2.16 ppm (s, 3 H; C*H*_3_); ^13^C NMR (125 MHz, DMSO): *δ*=173.5 (*C*=O), 166.4 (*C*‐4), 152.2 (*C*‐2), 150.8 (*C*‐6), 81.3 (*C*‐5), 23.0 ppm (CO*C*H_3_).

#### *N*‐(5‐{(2′*R*,5′*R*)‐4′‐[(*tert*‐Butyldimethylsilyl)oxy]‐5′‐{[(*tert*‐butyldimethylsilyl)oxy]methyl}‐2′,5′‐dihydrofuran‐2‐yl}‐6‐oxo‐1,6‐dihydropyrimidin‐2‐yl)acetamide (13)

*N*,*O*‐Bis(trimethylsilyl)‐acetamide (9 mL, 7.5 g, 37 mmol) was added dropwise to a suspension of **11** (8.0 g, 29 mmol) in DMF (50 mL) under an argon atmosphere. After stirring for 1 h the reaction become a clear solution. Then *N*,*N*‐diisopropylethylamine (6.3 mL, 4.67 g, 36 mmol) and 1,4‐anhydro‐3,5‐*O*‐bis(*tert*‐butyldimethylsilyl)‐2‐deoxy‐d‐*erythro*‐pent‐1‐enitol (**12**, 4.0 g, 11.6 mmol) were added. In a separate flask, Pd(OAc)_2_ (0.5 g, 2.2 mmol) was added to a solution of triphenylarsine (1.3 g, 4.2 mmol) in stirring DMF (100 mL). After 30 min, this solution was added slowly to the first flask and the mixture was stirred for 24 h at 80 °C. The reaction was quenched with the addition of H_2_O (30 mL) and the solvent was evaporated under reduced pressure. The residue was redissolved in EtOAc (500 mL), and washed with H_2_O (2×200 mL) and brine (200 mL). The organic layer was dried over MgSO_4_, filtered and concentrated under reduced pressure. The residue was purified by column chromatography on silica gel (EtOAc/hexane 7:3) to give **13** as a light yellow solid (2.8 g, 52 % yield). ^1^H NMR (500 MHz, CDCl_3_): *δ*=12.30 (br s, 1 H; N*H*), 9.86 (br s, 1 H; N*H*), 8.04 (s, 1 H; *H*‐6), 5.75 (s, 1 H; *H*‐1′), 4.92 (s, 1 H; *H*‐2′), 4.53–4.51 (m, 1 H; *H*‐4′), 3.84 (dd, *J=*11.5, 2.5 Hz, 1 H; *H*‐5′a), 3.69 (dd, *J=*11.5, 4.0 Hz, 1 H; *H*‐5′b), 0.92 (s, 9 H; C(C*H*_3_)_3_), 0.90 (s, 9 H; C(C*H*_3_)_3_), 0.09 (s, 6 H; Si(C*H*_3_)_2_), 0.02 ppm (s, 6 H; Si(C*H*_3_)_2_); ^13^C NMR (125 MHz, CD_3_OD): *δ*=174.2 (*C*=O), 161.3 (*C*‐4), 151.4 (*C*‐6), 148.1 (*C*‐2), 123.0 (*C*‐5), 101.3 (*C*‐2′), 85.4 (*C*‐4′), 77.9 (*C*‐1′) 64.7 (*C*‐5′), 26.5 (SiC(*C*H_3_)_3_), 26.1 (SiC(*C*H_3_)_3_), 22.5 (CO*C*H_3_), 19.4 (Si*C*(CH_3_)_3_), 18.8 (Si*C*(CH_3_)_3_), −6.1 (Si(*C*H_3_)_2_), −6.3 ppm (Si(*C*H_3_)_2_); MS (ES^+^): *m*/*z* (%): 496 [*M*+H]^+^ (40), 518.26 [*M*+Na]^+^ (100).

#### *N*‐{5‐[(2′*R*,4′*S*,5′*R*)‐4′‐Hydroxy‐5′‐(hydroxymethyl)tetrahydrofuran‐2′‐yl]‐6‐oxo‐1,6‐dihydropyrimidin‐2‐yl}acetamide (15)

70 % HF–pyridine (2.9 mL) was added dropwise to a solution of **13** (3.0 g, 6.0 mmol) in THF (100 mL). The reaction was stirred at room temperature for 12 h under an argon atmosphere. The suspension was diluted with acetic acid (30 mL) and the volatiles removed under reduced pressure to obtain crude compound **14**, which was used in the next step without further purification. ^1^H NMR (500 MHz, CD_3_OD): *δ*=8.1 (s, 1 H; *H*‐6), 5.17 (dd *J=*10.2, 6.8 Hz 1 H; *H*‐1′), 4.04 (t, *J=*3.5 Hz, 1 H; *H*‐4′), 3.65 (dd, *J=*12.2, 2.6 Hz, 1 H; *H*‐5′a), 3.61 (dd, *J=*12.2, 3.5 Hz, 1 H; *H*‐5′b), 2.89–2.86 (m, 1 H; *H*‐2′a), 2.53–2.50 (m, 1 H; *H*‐2′b), 2.23 ppm (s, 3 H; C*H*_3_); MS (ESI, negative‐ion mode): *m*/*z*: found 266.20 [*M*−H]^+^ 100 %; reversed‐phase HPLC, eluting with H_2_O/CH_3_CN from 98/2 to 0/100 in 45 min, flow=1 mL min^−1^, *λ*=254 nm, *t*_R_=7.75 min.

The residue was dissolved in a mixture of acetic acid/CH_3_CN (1:1 v/v, 200 mL) and the mixture was cooled to −15 °C, followed by the portionwise addition of NaBH(OAc)_3_ (3.0 g, 14.1 mmol). After 2 h, the mixture was evaporated to dryness under reduced pressure and the residue was purified by flash column chromatography (CH_2_Cl_2_/CH_3_OH 8:2) to give **15** as a white solid (1.3 g, 80 % yield). ^1^H NMR (500 MHz, [D_6_]DMSO): *δ*=12.09 (br s, 1 H; N*H*), 11.73 (br s, 1 H; N*H*), 8.1 (s, 1 H; *H*‐6), 5.14 (br s, 1 H; 5′‐O*H*), 4.95–4.90 (m, 1 H; *H*‐1′), 4.09 (t, *J=*3.3 Hz, 1 H; *H*‐4′), 3.65 (ddd, *J=*12.0, 4.2, 2.5 Hz, 1 H; *H*‐5′a), 3.61 (ddd, *J=*12.5, 7.3, 2.5 Hz, 1 H; *H*‐5′b), 3.17 (d, *J=*5.4 Hz, 1 H; 3′‐O*H*), 2.76 (dd, *J=*17.6, 6.6 Hz, 1 H; *H*‐2′a), 2.42 (dd, *J=*17.5, 10.0 Hz, 1 H; *H*‐2′b), 2.16 ppm (s, 3 H; C*H*_3_); ^13^C NMR (125 MHz, CD_3_OD): *δ*=175.1 (*C*=O), 159.6 (*C*‐4), 153.6 (*C*‐6), 152.7 (*C*‐2), 123.4 (*C*‐5), 88.9 (*C*‐4′), 75.7 (C‐1′), 73.4 (*C*‐3′), 64.1 (*C*‐5′), 41.7 (*C*‐2′), 23.9 ppm (*C*H_3_); MS (ES^+^): *m*/*z* (%): calcd for C_11_H_15_N_3_O_5_: 269 [*M*]; found: 291.09 [*M*+Na]^+^ (100); reversed‐phase HPLC, eluting with H_2_O/CH_3_OH from 90:10 to 0:100 in 30 min, flow=1 mL min^−1^, *λ*=254 nm, *t*_R_=4.87 min.

#### 1 ‐Amino‐5‐[(2′*R*,4′*S*,5′*R*)‐4′‐hydroxy‐5′‐(hydroxymethyl)tetrahydrofuran‐2′‐yl]pyrimidin‐4(3*H*)‐one (2)

Ammonia in MeOH (7 m, 1 mL) was added to a stirred solution of **15** (20.0 mg, 0.074 mmol) in MeOH (0.2 mL) at 0 °C under an argon atmosphere. After 30 min, the mixture was allowed to reach room temperature and was stirred for a further 6 h. The volatiles were removed under reduced pressure and the crude material was purified by preparative reversed‐phase HPLC (eluting with H_2_O/CH_3_OH from 100/0 to 0/100 over 30 min, flow rate 20 mL min^−1^) to give **2** as a white solid (12.0 mg, 71 % yield). ^1^H NMR (500 MHz, CD_3_OD): *δ*=7.68 (s, 1 H; *H*‐6), 5.00 (s, 1 H; *H*‐1′), 4.33 (d *J=*5.6 Hz, 1 H; *H*‐3′), 3.94–3.92 (m, 1 H; *H*‐4′), 3.83 (dd, *J=*12.2, 3.6 Hz, 1 H; *H*‐5′a), 3.63 (dd, *J=*12.7, 4.0 Hz, 1 H; *H*‐*5′*b), 2.23–2.17 (m, 1 H; *H*‐2′a), 2.10 ppm (dd, *J=*13.0, 6.0 Hz, 1 H; *H*‐2′b); ^13^C NMR (125 MHz, CD_3_OD): *δ*=166.1 (*C*‐4), 156.3 (C‐2), 148.6 (*C*‐6), 114.6 (*C*‐5), 87.5 (*C*‐4′), 76.0 (*C*‐1′), 73.4 (*C*‐3′), 62.6 (*C*‐5′), 40.3 ppm (*C*‐2′); MS (ES^+^): *m*/*z* (%): 228.04 [*M*+H]^+^ (50), 250.01 [*M*+Na]^+^ (100); reversed‐phase HPLC, eluting with H_2_O/CH_3_OH from 90:10 to 0:100 in 30 min, flow=1 mL min^−1^, *λ*=254 nm, *t*_R_=4.57 min.

#### *N*‐[5‐((2′*R*,4′*S*,5′*R*)‐4′‐[(*tert*‐Butyldimethylsilyl)oxy]‐5′‐{[(*tert*‐butyldimethylsilyl)oxy]methyl}tetrahydrofuran‐2′‐yl)‐6‐oxo‐1,6‐dihydropyrimidin‐2‐yl]acetamide (16)

Imidazole (0.4 g, 5.8 mmol), *tert*‐butyldimethylsilyl chloride (0.44 g, 2.9 mmol) and DMAP (0.04 g, 0.33 mmol) were added to a solution of **15** (0.34 g, 1.26 mmol) in DMF (2.5 mL) at room temperature under an argon atmosphere and the reaction was stirred at room temperature for 24 h. The reaction mixture was then quenched with CH_3_OH (1 mL) and the solvent was removed under reduced pressure. The residue was diluted with CH_2_Cl_2_ (5 mL) and washed with H_2_O (2 mL), NaHCO_3_ (2 mL) and brine (2 mL). The organic layer was dried over MgSO_4_, filtered and concentrated under reduced pressure. The crude product was purified by flash column chromatography (EtOAc/hexane 7:3) to give **16** as a white solid (0.410 g, 65 % yield). ^1^H NMR (500 MHz, CD_3_OD): *δ*=7.85 (s, 1 H; *H*‐6), 4.98 (dd, *J=*9.0, 5.5 Hz, 1 H; *H*‐1′), 4.32–4.30 (m, 1 H; *H*‐3′), 3.78–3.76 (m, 1 H; *H*‐4′), 3.64 (dd, *J=*10.5, 4.5 Hz, 1 H; *H*‐5′a), 3.56 (dd, *J=*10.5, 4.5 Hz, 1 H; *H*‐5′b), 2.21–2.16 (m, 1 H; *H*‐2′a), 2.10 (s, 3 H; C*H*_3_), 1.79–1.69 (m, 1 H; *H*‐2′b), 0.83 (s, 9 H; C(C*H*_3_)_3_), 0.81 (s, 9 H; C(C*H*_3_)_3_), 0.02 (s, 3 H; Si(C*H*_3_)_2_), 0.02 (s, 3 H; Si(C*H*_3_)_2_), 0.01 (s, 3 H; Si(C*H*_3_)_2_), 0.00 ppm (s, 3 H; Si(C*H*_3_)_2_); ^13^C NMR (125 MHz, CD_3_OD): *δ*=173.6 (*C*=O), 159.6 (*C*‐4), 151.0 (*C*‐6), 148.4 (*C*‐2), 123.1 (*C*‐5), 87.6 (*C*‐4′), 74.1 (*C*‐1′, *C*‐3′), 63.4 (*C*‐5′), 40.9 (*C*‐2′), 25.1 (C(*C*H_3_)_3_), 25.0 (C(*C*H_3_)_3_), 22.5 (CO*C*H_3_), 17.8 (*C*(CH_3_)_3_), 17.5 (*C*(CH_3_)_3_), −5.8 (Si(*C*H_3_)_2_), −5.9 (Si(*C*H_3_)_2_), −6.6 (Si(*C*H_3_)_2_), −6.7 ppm (Si(*C*H_3_)_2_); MS (ES^+^): *m*/*z* (%): 498.3 [*M*+H]^+^ (50), 519.2 [*M*+Na]^+^ (100).

#### *N*‐(5‐{(2′*R*,4′*S*,5′*R*)‐4′‐[(*tert*‐Butyldimethylsilyl)oxy]‐5′‐(hydroxymethyl)tetrahydrofuran‐2′‐yl}‐6‐oxo‐1,6‐dihydropyrimidin‐2‐yl)acetamide (17)

A mixture of TFA and H_2_O (1:1 v/v, 2.4 mL) was added dropwise to a solution of **16** (0.3 g, 0.6 mmol) in THF (4.8 mL) at 0 °C. The reaction mixture was stirred at room temperature for 2 h under an argon atmosphere, then quenched with aqueous NaHCO_3_; CH_2_Cl_2_ was added and the aqueous phase was extracted twice with CH_2_Cl_2_. The combined organic layers were dried over MgSO_4_, filtered and concentrated under vacuum to yield **17** as a glassy solid (0.172 g, 75 %), which was used in the next step without further purification. ^1^H NMR (500 MHz, CD_3_OD): *δ*=7.92 (s, 1 H; *H*‐6), 4.94 (dd, *J=*9.0, 5.5 Hz, 1 H; *H*‐1′), 4.45–4.42 (m, 1 H; *H*‐3′), 3.75 (dt, *J=*4.5, 2.5 Hz, 1 H; *H*‐4′), 3.68 (dd, *J=*11.9, 4.5 Hz, 1 H; C*H*_2a_‐5′), 3.66 (dd, *J=*11.9, 4.5 Hz, 1 H; C*H*_2b_‐5′), 2.30–2.14 (m, 1 H; C*H*_2a_‐2′), 2.22 (s, 3 H; C*H*_3_), 2.12–1.99 (m, 1 H; C*H*_2b_‐2′), 0.95 (s, 9 H; C(C*H*_3_)_3_), 0.02 (s, 3 H; Si(C*H*_3_)_2_), 0.01 ppm (s, 3 H; Si(C*H*_3_)_2_); ^13^C NMR (125 MHz, CD_3_OD): *δ*=173.5 (*C*=O), 160.3 (*C*‐4), 149.9 (*C*‐6), 148.2 (*C*‐2), 123.0 (*C*‐5), 87.5 (*C*‐4′), 83.7 (*C*‐1′), 74.2 (*C*‐3′), 71.0 (*C*‐5′), 39.4 (*C*‐2′), 25.0 (SiC(*C*H_3_)_3_), 22.5 (CO*C*H_3_), 17.7 (Si*C*(CH_3_)_3_), −5.9 (Si(*C*H_3_)_2_), −6.6 ppm (Si(*C*H_3_)_2_); MS (ES^+^): *m*/*z* (%): 384.2 [*M*+Na]^+^ (100); reversed‐phase HPLC, eluting with H_2_O/CH_3_OH from 90:10 to 0:100 in 30 min, flow=1 mL min^−1^, *λ*=254 nm, *t*_R_=16.37 min.

#### Standard Procedure 1: Synthesis of Phosphorochloridates 18 a–g

Anhydrous triethylamine (2.0 mol equiv.) was added dropwise at −78 °C to a stirred solution of the appropriate amino ester hydrochloride/tosylate salt (1.0 mol equiv.) and the appropriate dichlorophosphate (1.0 mol equiv.) in anhydrous dichloromethane (61.6 mol) under an argon atmosphere. After 1 h the reaction mixture was allowed to warm to room temperature and was stirred for an additional 1–2 h. Formation of the desired phosphorochloridate was monitored by ^31^P NMR spectroscopy. After the reaction was completed, the solvent was removed under reduced pressure and the resulting residue was re‐dissolved in anhydrous diethyl ether and the triethylammonium salt was removed by filtration. The filtrate was evaporated to dryness and the crude material was purified by flash column chromatography with ethyl acetate/hexane (1:1 v/v) as the eluent to give the desired phosphorochloridate as an oil.

#### Phenyl‐(benzoxy‐l‐alaninyl)‐phosphorochloridate (18 a)

Prepared according to standard procedure 1 in 92 % yield. ^1^H NMR (500 MHz, CDCl_3_): *δ*=7.30–7.10 (m, 10 H; *H*‐Ar), 5.20–5.16 (m, 2 H; OC*H*_2_Ph), 4.25–4.22 (m, 1 H; C*H*CH_3_), 3.51–3.48 (m, 1 H; N*H*), 1.54 (d, *J=*7.3 Hz, 1.5 H; CHC*H*_3_), 1.52 ppm (d, *J=*7.3 Hz, 1.5 H; CHC*H*_3_); ^13^C NMR (125 MHz, CDCl_3_): *δ*=169.8 (d, *J*_*C–P*_=5.4 Hz; *C*=O), 135.0 (d, *J*_*C‐P*_=6.8 Hz; *ipso*‐*C*‐Ph), 134.7 (*C‐ipso*‐OCH_2_Ph), 130.0, 129.8 129.6, 128.7, 128.7, 128.4, 128.3, 126.0 (*C*H‐Ph), 120.6 (d, *J*_*C–P*_=2.5 Hz; *C*H‐Ph), 120.5 (d, *J*_*C–P*_=2.5 Hz; *C*H‐Ph), 68.1, 67.7 (O*C*H_2_Ph), 50.8, 50.5 (*C*HCH_3_), 20.5 ppm (d, *J=*5.6 Hz; CH*CH*_3_); ^31^P NMR (202 MHz, CDCl_3_): *δ*=7.93 (0.5 P), 7.51 ppm (0.5 P).

#### Phenyl‐(hexoxy‐l‐alaninyl)‐phosphorochloridate (18 b)

Prepared according to standard procedure 1 in 87 % yield. ^1^H NMR (500 MHz, CDCl_3_): *δ*=7.24–7.18 (m, 5 H; *H*‐Ph), 4.34–4.20 (m, 1 H; N*H*), 4.20–4.05 (m, 3 H; C*H*CH_3_ and OC*H*_2_), 4.03–3.94 (m, 2 H; OCH_2_C*H*_2_), 1.66–1.56 (m, 4 H; OCH_2_CH_2_C*H*_2_C*H*_2_), 1.59–1.53 (m, 3 H; CHC*H*_3_), 1.37–1.31 (m, 4 H; C*H*_2_C*H*_2_CH_3_), 0.94–0.87 ppm (CH_2_C*H*_3_); ^13^C NMR (125 MHz, CDCl_3_): *δ*=172.8 (d, *J*_*C–P*_=7.8 Hz; *C*=O), 172.7 (d, *J*_*C–P*_=7.8 Hz; *C*=O), 149.8 (d, *J*_*C–P*_=8.0 Hz; *ipso*‐*C*‐Ph), 149.8 (d, *J*_*C–P*_=8.0 Hz; *ipso*‐*C*‐Ph), 129.9, 129.8, 125.9, 125.9 (*C*H‐Ph), 120.6 (d, *J*_*C–P*_=5.3 Hz; *C*H‐Ph), 66.0 65.9 (O*C*H_2_), 50.8, 50.5 (*C*HCH_3_), 31.3 (OCH_2_ *C*H_2_), 28.4 (OCH_2_CH_2_ *C*H_2_), 25.4 (OCH_2_CH_2_CH_2_ *C*H_2_), 22.5 (*C*H_2_CH_3_), 22.4 (d, *J*_*C–P*_ *=*5.6 Hz; CH*CH*_3_), 22.3 (d, *J*_*C–P*_ *=*5.6 Hz; CH*CH*_3_), 13.9 ppm (CH_2_ *C*H_3_); ^31^P NMR (202 MHz, CDCl_3_): *δ*=7.96 (0.5 P), 7.64 ppm (0.5 P).

#### Phenyl‐(pentoxy‐l‐alaninyl)‐phosphorochloridate (18 c)

Prepared according to standard procedure 1 in 96 % yield. ^1^H NMR (500 MHz, CDCl_3_): *δ*=7.46–7.31 (m, 2 H; *H*‐Ph), 7.28–7.22 (m, 3 H; *H*‐Ph), 4.68 (br s; N*H*), 4.18–4.09 (m, 3 H; OC*H*_2_, C*H*CH_3_), 1.73–1.71 (m, 2 H; OCH_2_C*H*_2_), 1.68–1.65 (m, 5 H; OCH_2_CH_2_C*H*_2_, CHC*H*_3_), 1.36–1.32 (m, 2 H; C*H*_2_CH_3_), 0.92–0.89 ppm (m, 3 H; C*H*_3_); ^13^C NMR (125 MHz, CDCl_3_): *δ*=172.7 (d, *J*_*C–P*_=7.7 Hz; *C*=O), 172.6 (d, *J*_*C–P*_=7.3 Hz; *C*=O), 149.6 (d, *J*_*C–P*_=8.1 Hz; *ipso*‐*C*‐Ph), 149.4 (d, *J*_*C–P*_=8.0 Hz; *ipso*‐*C*‐Ph), 129.9, 129.8, 125.9, 125.8 (*C*H‐Ph), 120.5 (d, *J*_*C–P*_=5.5 Hz, *C*H‐Ph), 65.9 65.8 (O*C*H_2_), 50.5, 50.3 (*C*HCH_3_), 31.3 (OCH_2_ *C*H_2_), 28.3 (OCH_2_CH_2_ *C*H_2_), 22.4 (*C*H_2_CH_3_), 22.4 (d, *J=*5.8 Hz, CH*CH*_3_), 22.4 (d, *J=*5.7 Hz, CH*CH*_3_), 13.9 ppm (7 CH_2_ *C*H_3_); ^31^P NMR (202 MHz, CDCl_3_): *δ*=7.92 (0.5 P), 7.61 ppm (0.5 P).

#### 2 ‐Naphthyl‐(isopropoxy‐l‐alaninyl)‐phosphorochloridate (18 d)

Prepared according to standard procedure 1 in 84 % yield. ^1^H NMR (500 MHz, CDCl_3_): *δ*=8.10–8.07 (m, 1 H; *CH*‐Naph), 7.88–7.82 (m, 1 H; *CH*‐Naph), 7.76–7.56 (m, 1 H; *H*‐Naph), 7.6–7.3 (m, 4 H; *H*‐Naph), 5.13–5.09 (m, 1 H; OC*H*(CH_3_)_2_), 4.54 (br s, 1 H; N*H*), 4.26–4.22 (m, 1 H; C*H*CH_3_), 1.56 (d, *J=*7.0 Hz, 1.5 H; CHC*H*_3_), 1.54 (d, *J=*7.0 Hz, 1.5 H; CHC*H*_3_), 1.34–1.25 ppm (m, 6 H; OCH(C*H*_3_)_2_); ^13^C NMR (125 MHz, CDCl_3_): *δ*=173.1 (*C*=O), 149.8 (d, *J*_*C–P*_=8.0 Hz; *ipso*‐*C*‐Ph), 147.3 (*ipso*‐*C*‐Ph), 134.9, 134.8 (*C*‐Naph), 129.0, 127.6, 126.9, 126.7, 126.3 (*C*H‐Naph), 126.1 (d, *J*_*C–P*_=8.3 Hz; *C*‐Naph), 125.5, 124.4, 122.0, 121.5, 121.4, 121.3, 116.2, 116.1, 115.2 (*C*H‐Naph), 70.6, 69.2 (O*C*H(CH_3_)_2_), 51.0, 50.7 (*C*HCH_3_), 21.7, 21.5 (CH*C*H_3_), 16.1 ppm (OCH(*C*H_3_)_2_); ^31^P NMR (202 MHz, CDCl_3_): *δ*=8.35 (0.5 P), 8.03 ppm (0.5 P).

#### 3 ‐Naphthyl‐(2,2‐dimethyl‐propoxy‐l‐alaninyl)‐phosphorochloridate (18 e)

Prepared according to standard procedure 1 in 91 % yield. ^1^H NMR (500 MHz, CDCl_3_): *δ*=8.20–7.30 (m, 7 H; *H*‐Naph), 4.48–4.34 (br s, 1 H; N*H*), 4.38–4.20 (m, 1 H; C*H*CH_3_), 3.90–3.70 (m, 2 H; C*H*_2_C(CH_3_)_3_), 1.58–1.45 (m, 3 H; CHC*H*_3_), 0.89 (s, 4.5 H; C(C*H*_3_)_3_), 0.87 ppm (s, 4.5 H; C(C*H*_3_)_3_); ^13^C NMR (125 MHz, CDCl_3_): *δ*=171.7 (d, *J*_*C–P*_=2.5 Hz; *C*=O), 171.6, (d, *J*_*C–P*_=2.5 Hz; *C*=O), 146.7 (d, *J*_*C–P*_=6.3 Hz; *ipso*‐*C*‐Naph), 134.7 (*C*‐Naph), 128.9, 129.0, 127.9, 126.9, 126.8, 126.2 (*C*H‐Naph), 126.1 (d, *J*_*C–P*_=8.7 Hz; *C*‐Naph), 125.9, 121.4, 121.3 (*C*H‐Naph), 75.2 (*C*H_2_C(*C*H_3_)_3_), 75.1 (*C*H_2_C(*C*H_3_)_3_), 50.9 (*C*HCH_3_), 50.6 (*C*HCH_3_), 26.4 (C(*C*H_3_)_3_), 26.3 (C(*C*H_3_)_3_), 20.9 ppm (d, *J=*4.8 Hz, CH*CH*_3_); ^31^P NMR (202 MHz, CDCl_3_): *δ*=8.25 (0.5 P), 7.95 ppm (0.5 P).

#### 4 ‐Naphthyl‐(benzoxy‐l‐alaninyl)‐phosphorochloridate (18 f)

Prepared according to standard procedure 1 in 78 % yield. ^1^H NMR (500 MHz, CDCl_3_): *δ*=8.12–7.97 (m, 1 H; *H*‐Naph), 7.73–7.57 (m, 1 H; *H*‐Naph), 7.65–7.32 (m, 10 H; *H*‐Ar), 5.25–5.21 (m, 2 H; OC*H*_2_Ph), 4.81–4.78 (m, 1 H; N*H*), 4.23–4.20 (m, 1 H; C*H*CH_3_), 1.59–1.57 ppm (m, 3 H; CHC*H*_3_); ^13^C NMR (125 MHz, CDCl_3_): *δ*=173.9 (d, *J*_*C–P*_=6.5 Hz; *C*=O), 173.8 (d, *J*_*C–P*_=6.5 Hz; *C*=O), 146.8 (d, ^2^ *J*_*C–P*_=6.3 Hz; *ipso*‐*C*‐Naph), 135.4, 134.8 (*C*‐Naph), 128.6, 128.4, 128.2, 128.1, 126.7, 126.5, 126.3, 126.0 (d, *J*_*C–P*_=8.2 Hz; *C*‐Naph), 125.7, 124.4, 121.5, 115.2, 115.2 (*C*H‐Ph, *C*H‐Naph), 67.1 (O*C*H_2_Ph), 50.2 (*C*HCH_3_), 50.1 (d, ^3^ *J*_*C–P*_=2.0 Hz; *C*HCH_3_), 21.1 (d, *J=*5.3 Hz; CH*CH*_3_), 21.0 ppm (d, *J=*5.3 Hz; CH*CH*_3_); ^31^P NMR (202 MHz, CDCl_3_): *δ*=8.19 (0.5 P), 7.94 ppm (0.5 P).

#### Phenyl‐(isopropoxy‐l‐alaninyl)‐phosphorochloridate (18 g)

Prepared according to standard procedure 1 in 93 % yield. ^1^H NMR (500 MHz, CDCl_3_): *δ*=7.41–7.36 (m, 2 H; *H*‐Ph), 7.31–7.27 (m, 3 H; *H*‐Ph), 5.21–5.01 (m, 1 H; OC*H*(CH_3_)_2_), 4.65 (br s, 1 H; N*H*), 4.21–4.06 (m, 1 H; C*H*CH_3_), 1.51 (d, *J=*7.0 Hz, 3 H; CHC*H*_3_), 1.26–1.19 ppm (m, 6 H; OCH(C*H*_3_)_2_); ^13^C NMR (125 MHz, CD_3_OD): *δ*=172.2 (d, *J*_*C–P*_=8.2 Hz; *C*=*O*), 172.1 (d, *J*_*C–P*_=9.1 Hz; *C*<C=*>O*), 149.8 (d, *J*_*C–P*_=8.4 Hz; *ipso*‐*C*‐Ph), 149.8 (d, *J*_*C–P*_=8.3 Hz; *ipso*‐*C*‐Ph), 129.9 (*C*H‐Ph), 126.0 (*C*H‐Ph), 120.5 (d, *J*_*C–P*_=5.4 Hz; *C*H‐Ph), 69.8, 69.8 (O*C*H(CH_3_)_2_), 50.9, 50.6 (*C*HCH_3_), 21.6, 21.5 (OCH(*C*H_3_)_2_), 20.4 (d, *J*_*C–P*_=4.3 Hz; CH*C*H_3_), 20.6 ppm (d, *J*_*C–P*_=4.3 Hz; CH*C*H_3_); ^31^P NMR (202 MHz, CDCl_3_): *δ*=8.08 (0.5 P), 7.71 ppm (0.5 P).

#### Standard Procedure 2: Synthesis of 20 a–f, 24 a and 24 g

A solution of *t*BuMgCl in THF (1.0 m, 1.2 mol equiv.) was added at 0 °C to a stirred solution of **17** or **23** (1 mol equiv.) in THF. The reaction mixture was allowed to warm to room temperature and the appropriate phosphorochloridate (2.0 mol equiv.) dissolved in anhydrous THF was added. The reaction mixture was stirred for 16–18 h and then evaporated under vacuum to give a crude residue that was purified by column chromatography on silica gel, eluting with a gradient of CH_3_OH (0–5 %) in CH_2_Cl_2_ to afford products **20 a**–**f**, **24 a** and **24 g** as white solids.

#### (2*S*)‐Benzyl 2‐{[({(2′*R*,3′*S*,5′*R*)‐5‐(2*‐*Acetamido‐6‐oxo‐1,6‐dihydropyrimidin‐5‐yl)‐3′‐[(*tert*‐butyldimethylsilyl)oxy]tetrahydrofuran‐2′‐yl}methoxy)(phenoxy)phosphoryl]amino}propanoate (20 a)

Prepared according to standard procedure 2 from nucleoside **17** (0.048 g, 0.137 mmol), **18 a** (0.088 g, 0.27 mmol) in anhydrous THF (2.4 mL) and *t*BuMgCl in THF (1 m, 0.16 mL). After workup, the crude product was purified by column chromatography on silica gel using CH_2_Cl_2_/CH_3_OH (95:5) as eluent to provide **20 a** as a white solid (22 mg, 25 % yield). ^1^H NMR (500 MHz, CD_3_OD): *δ*=7.89 (s, 1 H; *H*‐6), 7.35–7.32 (m, 7 H; *H*‐Ar), 7.22–7.14 (m, 3 H; *H*‐Ar), 5.18–5.00 (m, 3 H; *H*‐1′, OC*H*_2_Ph), 4.40–4.39 (m, 1 H; *H*‐3′), 4.21–4.12 (m, 2 H; C*H_2_*‐5′), 4.05–4.00 (m, 2 H; *H*‐4′, C*H*CH_3_), 2.33–2.23 (m, 1 H; C*H_2a_*‐2′), 2.21 (s, 1.2 H; COC*H*_3_), 2.20 (s, 1.8 H; COC*H*_3_), 1.81–1.71 (m, 1 H; C*H_2b_*‐2′) 1.37 (d, *J=*7.1 Hz, 1.2 H; CHC*H*_3_), 1.36 (d, *J=*7.2 Hz, 1.2 H; CHC*H*_3_), 0.92 (s, 9 H; C(C*H*_3_)_3_), 0.11 (s, 1.5 H; Si(C*H*_3_)_2_), 0.10, (s, 1.5 H; Si(C*H*_3_)_2_), 0.09 ppm (s, 3 H; Si(C*H*_3_)_2_); ^31^P NMR (202 MHz, CD_3_OD): *δ*=3.89 (0.6 P), 3.48 ppm (0.4 P); MS (ES^+^): *m*/*z* (%): 701 [*M*+H]^+^ (30), 723.30 [*M*+Na]^+^ (100).

#### (2*S*)‐Hexyl 2‐{[({(2′*R*,3′*S*,5′*R*)‐5‐(2*‐*Acetamido‐6‐oxo‐1,6‐dihydropyrimidin‐5‐yl)‐3‐[(*tert*‐butyldimethylsilyl)oxy]tetrahydrofuran‐2‐yl}methoxy)(phenoxy)phosphoryl]amino}propanoate (20 b)

Prepared according to standard procedure 2 from nucleoside **17** (0.090 g, 0.256 mmol), **18 b** (0.177 g, 0.512 mmol) in anhydrous THF (2.4 mL) and *t*BuMgCl in THF (1 m, 0.30 mL). After workup, the crude was purified by column chromatography on silica gel with CH_2_Cl_2_/CH_3_OH (95:5) as eluent to provide **20 b** as a solid (34 mg, 21 % yield). ^1^H NMR (500 MHz, CD_3_OD): *δ*=7.90 (s, 1 H; *H*‐6), 7.36–7.31 (m, 2 H; *H*‐Ph), 7.24–7.15 (m, 3 H; *H*‐Ph), 5.11–5.04 (m, 1 H; *H*‐1′), 4.44–4.42 (m, 1 H; *H*‐3′), 4.26–4.17 (m, 2 H; C*H_2_*‐5′), 4.10–4.13 (m, 2 H; OC*H*_2_), 4.04–4.03 (m, 1 H; *H*‐4′), 4.00–3.94 (m, 1 H; C*H*CH_3_), 2.33–2.26 (m, 1 H; C*H_2a_*‐2′), 2.23 (s, 1.5 H; COC*H*_3_), 2.22 (s, 1.5 H; COC*H*_3_), 1.86–1.78 (m, 1 H; C*H_2b_*‐2′), 1.70–1.63 (OCH_2_ *C*H_2_), 1.38–1.28 (m, 9 H; (C*H*_2_)_3_CH_3_, CHC*H*_3_), 0.93 (s, 4.5 H; C(C*H*_3_)_3_), 0.92 (s, 4.5 H; C(C*H*_3_)_3_), 0.92–0.88 (m, 3 H; CH_2_C*H*_3_), 0.11 (s, 1.5 H; Si(C*H*_3_)_2_), 0.11, (s, 1.5 H; Si(C*H*_3_)_2_), 0.10 ppm (s, 3 H; Si(C*H*_3_)_2_); ^31^P NMR (202 MHz, CD_3_OD): *δ*=3.80 (0.6 P), 3.48 ppm (0.4 P); MS (ES^+^): *m*/*z* (%): 695.85 [*M*+H]^+^ (30), 717.50 [*M*+Na]^+^ (100).

#### (2*S*)‐Pentyl 2‐{[({(2′*R*,3′*S*,5′*R*)‐5‐(2*‐*Acetamido‐6‐oxo‐1,6‐dihydropyrimidin‐5‐yl)‐3‐[(*tert*‐butyldimethylsilyl)oxy]tetrahydrofuran‐2‐yl}methoxy)(phenoxy)phosphoryl]amino}propanoate (20 c)

Prepared according to standard procedure 2 from nucleoside **17** (0.100 g, 0.284 mmol), **18 c** (0.189 g, 0.568 mmol) in anhydrous THF (2.6 mL) and *t*BuMgCl in THF (1 m, 0.33 mL). After workup, the crude material was purified by column chromatography on silica gel with CH_2_Cl_2_/CH_3_OH (95:5) as the eluent to provide **20 c** as a solid (50 mg, 28 % yield). ^1^H NMR (500 MHz, CD_3_OD): *δ*=7.88 (s, 1 H; *H*‐6), 7.33–7.28 (m, 2 H; *H*‐Ph), 7.21–7.12 (m, 3 H; *H*‐Ph), 5.14–5.06 (m, 1 H; *H*‐1′), 4.40–4.38 (m, 1 H; *H*‐3′), 4.25–4.16 (m, 2 H; C*H_2_*‐5′), 4.13–4.10 (m, 2 H; OC*H*_2_), 4.03–4.00 (m, 1 H; *H*‐4′), 4.00–3.94 (m, 1 H; C*H*CH_3_), 2.35–2.28 (m, 1 H; C*H_2a_*‐2′), 2.25 (s, 3 H; COC*H*_3_), 1.86–1.75 (m, 1 H; C*H_2b_*‐2′), 1.73–1.70 (m, 2 H; OCH_2_C*H*_2_), 1.69–1.65 (m, 5 H; OCH_2_CH_2_C*H*_2_, CHC*H*_3_), 1.36–1.32 (m, 2 H; C*H*_2_CH_3_), 0.94 (s, 4.5 H; C(C*H*_3_)_3_), 0.92 (s, 4.5 H; C(C*H*_3_)_3_), 0.91–0.89 (m, 3 H; CH_2_ *C*H_3_), 0.11 (s, 1.5 H; Si(C*H*_3_)_2_), 0.11, (s, 1.5 H; Si(C*H*_3_)_2_), 0.10 ppm (s, 3 H; Si(C*H*_3_)_2_); ^31^P NMR (202 MHz, CD_3_OD): *δ*=3.85 (0.5 P), 3.56 ppm (0.5 P); MS (ES^+^): *m*/*z* (%): 681.8 [*M*+H]^+^ (30), 703.50 [*M*+Na]^+^ (100).

#### (2*S*)‐Isopropyl 2‐{[({(2′*R*,3′*S*,5′*R*)‐5‐(2‐Acetamido‐6‐oxo‐1,6‐dihydropyrimidin‐5‐yl)‐3′‐[(*tert*‐butyldimethylsilyl)oxy]tetrahydrofuran‐2′‐yl}methoxy)(naphthalen‐1‐yloxy)phosphoryl]amino}propanoate (20 d)

Prepared according to standard procedure 2 from nucleoside **17** (0.180 g, 0.47 mmol), **18 d** (0.335 g, 0.94 mmol) in anhydrous THF (2.4 mL) and *t*BuMgCl in THF (1 m, 0.56 mL). After workup, the crude material was purified by column chromatography on silica gel with CH_2_Cl_2_/CH_3_OH (95:5) as the eluent to provide **20 d** as a solid (40 mg, 12 % yield). ^1^H NMR (500 MHz, CD_3_OD): *δ*=8.13–8.10 (m, 1 H; *H*‐Naph), 7.85–7.81 (m, 2 H; *H*‐Naph, *H*‐6), 7.71–7.67 (m, 1 H; *H*‐Naph), 7.56–7.15 (m, 4 H; *H*‐Naph), 5.10–4.90 (m, 2 H; *H*‐1′, OC*H*(CH_3_)_2_), 4.25–4.10 (m, 3 H; C*H_2_*‐5′, *H*‐3′), 4.05–3.94 (m, 2 H; C*H*CH_3_, *H*‐4′), 2.26–2.15 (m, 1 H; C*H_2a_*‐2′), 2.23 (s, 3 H; COC*H*_3_), 2.01–1.98 (m, 1 H; C*H_2b_*‐2′), 1.38–134 (m, 3 H; CHC*H*_3_), 1.21 (s, 6 H; CH(C*H*_3_)_2_), 0.86 (s, 9 H; C(C*H*_3_)_3_), 0.018 (s, 3 H; Si(C*H*_3_)_2_), 0.00 ppm (s, 3 H; Si(C*H*_3_)_2_); ^31^P NMR (202 MHz, CD_3_OD): *δ*=4.31 ppm (1P); MS (ES^+^): *m*/*z* (%): 703.40 [*M*+H]^+^ (30), 725.40 [*M*+Na]^+^ (100); reversed‐phase HPLC, eluting with H_2_O/CH_3_CN from 90:10 to 0:100 in 30 min, flow=1 mL min^−1^, *λ*=254 nm, *t*_R_=24.85 min.

#### (2*S*)‐Neopentyl 2‐{[({(2′*R*,3′*S*,5′*R*)‐5‐(2‐Acetamido‐6‐oxo‐1,6‐dihydropyrimidin‐5‐yl)‐3′‐[(*tert*‐butyldimethylsilyl)oxy]tetrahydrofuran‐2′‐yl}methoxy)(naphthalen‐1‐yloxy)phosphoryl]amino}propanoate (20 e)

Prepared according to standard procedure 2 from nucleoside **17** (0.172 g, 0.45 mmol), **18 e** (0.345 g, 0.9 mmol) in anhydrous THF (2.3 mL) and *t*BuMgCl in THF (1 m, 0.54 mL). After workup, the crude material was purified by column chromatography on silica gel with CH_2_Cl_2_/CH_3_OH (94:6) as the eluent to provide **20 e** as a solid (42.0 mg, 13 % yield). ^1^H NMR (500 MHz, CD_3_OD): δ=7.94 (d, *J=*8.6 Hz, 0.5 H; *H*‐Naph), 7.82 (d, *J=*8.6 Hz, 0.5 H; *H*‐Naph), 7.78–7.63 (m, 2 H; *H*‐Naph, *H*‐6), 7.50–7.21 (m, 5 H; *H*‐Naph), 4.98 (dd, *J=*10.0, 5.5 Hz, 0.5 H; *H*‐1′), 4.90 (dd, *J=*10.0, 5.5 Hz, 0.5 H; *H*‐1′), 4.26–4.21 (m, 1 H; *H*‐3′), 4.12–4.06 (m, 2 H; C*H_2_*‐5′), 3.75–3.71 (m, 1 H; C*H*CH_3_), 3.70–3.58 (m, 2 H; C*H*_2_C(C*H*_3_)_3_), 3.39–3.31 (m, 1 H; *H*‐4′), 2.12 (s, 1.5 H; COC*H*_3_), 2.13–2.08 (m, 1 H; C*H_2a_*‐2′), 2.09 (s, 1.5 H; COC*H*_3_), 2.03–1.98 (m, 1 H; C*H_2b_*‐2′), 1.28 (d, *J=*7.0 Hz, 1.5 H; CHC*H*_3_), 1.25 (d, *J=*7.0 Hz, 1.5 H; CHC*H*_3_), 0.81 (s, 4.5 H; C(C*H*_3_)_3_), 0.79 (s, 4.5 H; C(C*H*_3_)_3_), 0.00 (s, 3 H; Si(C*H*_3_)_2_), −0.031 (s, 1.5 H; Si(C*H*_3_)_2_), −0.039 ppm (s, 1.5 H; Si(C*H*_3_)_2_); ^31^P NMR (202 MHz, CD_3_OD): *δ*=4.30 (0.5 P), 3.91 ppm (0.5 P); MS (ES^+^): *m*/*z* (%): 731.32 [*M*+H]^+^ (30), 753.30 [*M*+Na]^+^ (100); reversed‐phase HPLC, eluting with H_2_O/CH_3_CN from 90:10 to 0:100 in 30 min, flow=1 mL min^−1^, *λ*=254 nm, *t*_R_=17.93 min.

#### (2S)‐Benzyl {[((2′*R*,3′*S*,5′*R*)‐5‐{2‐Acetamido‐4‐[({[(*S*)‐1‐(benzyl‐ oxy)‐1‐oxopropan‐2‐yl]amino}(naphthalen‐1‐yloxy)phosphoryl)oxy]pyrimidin‐5‐yl}‐3′‐[(*tert*‐butyldimethylsilyl)oxy]tetrahydrofuran‐2′‐yl)methoxy](naphthalen‐1‐yloxy)phosphoryl}‐l‐alaninate (19 f) and (2*S*)‐Benzyl 2‐{[({(2′*R*,3′*S*,5′*R*)‐5‐(2‐Acetamido‐6‐oxo‐1,6‐dihydropyrimidin‐5‐yl)‐3′‐[(*tert*‐butyldimethylsilyl)oxy]tetrahydrofuran‐2′‐yl}methoxy)(naphthalen‐1‐yloxy)phosphoryl]amino}propanoate (20 f)

Prepared according to standard procedure 2 from nucleoside **17** (0.095 g, 0.248 mmol), **18 f** (0.200 g, 0.495 mmol) in anhydrous THF (4 mL) and *t*BuMgCl in THF (1 m, 0.37 mL). After workup, the crude material was purified by column chromatography on silica gel with CH_2_Cl_2_/CH_3_OH (98:2) as the eluent to provide **19 f** as a solid (56 mg, 21 % yield). ^1^H NMR (500 MHz, CDCl_3_): *δ*=8.90 (br s, 1 H; N*H*), 8.88 (br s, 1 H; NH), 8.55 (s, 0.3 H; *H*‐6), 8.49 (s, 0.3 H; *H*‐6), 8.44 (s, 0.4 H; *H*‐6), 7.81–7.17 (m, 22 H; *H*‐Naph, *H*‐Ph), 5.14–4.94 (m, 5 H; 2×OC*H*_2_Ph, *H*‐1′), 4.47–3.94 (m, 5 H; *H*‐3′, C*H_2_*‐5′, 2×C*H*CH_3_), 3.78–3.79 (m, 1 H; *H*‐4′), 2.22 (s, 1.5 H; COC*H*_3_), 2.18 (s, 0.8 H; COC*H*_3_), 2.22 (s, 0.7 H; COC*H*_3_), 2.08–2.013 (m, 0.5 H; C*H_2_*‐2′), 1.88–1.86 (m, 0.5 H; C*H_2_*‐2′), 1.79–1.74 (m, 0.5 H; C*H_2_*‐2′), 1.65–1.59 (m, 0.5 H; C*H_2_*‐2′), 0.90 (s, 2.3 H; C(C*H*_3_)_3_), 0.88 (s, 2.3 H; C(C*H*_3_)_3_), 0.87 (s, 4.6 H; C(C*H*_3_)_3_), 0.07 (s, 1 H; Si(C*H*_3_)_2_), 0.05 (s, 1 H; Si(C*H*_3_)_2_), 0.15 (s, 1 H; Si(C*H*_3_)_2_), 0.00 (s, 2 H; Si(C*H*_3_)_2_), −0.02 ppm (s, 1 H; Si(C*H*_3_)_2_); ^31^P NMR (202 MHz, CD_3_OD): δ=3.96 (0.25 P), 3.81 (0.25 P), 3.25 (0.5 P), 3.19 (0.7 P), 3.06 ppm (0.3 P); MS (ES^+^): *m*/*z* (%): 1120.32 [*M*+H]^+^ (60), 1142.30 [*M*+Na]^+^ (100).

Further elution of the crude mixture with CH_2_Cl_2_/CH_3_OH (95:5) yielded **20 f** as a solid (98 mg, 59 % yield). ^1^H NMR (500 MHz, CD_3_OD): *δ*=8.10 (d, *J=*8.6 Hz, 1 H; *H*‐Naph), 7.87 (d, *J=*8.6 Hz, 1 H; *H*‐Naph), 7.72–7.62 (m, 2 H, *H*‐Naph; *H*‐6), 7.53–7.39 (m, 4 H; *H*‐Naph), 7.32–7.27 (m, 5 H; *H*‐Ph), 5.12–5.05 (m, 2 H; OC*H*_2_Ph), 4.98 (dd, *J=*10.0, 5.5 Hz, 1 H; *H*‐1′), 4.26–4.14 (m, 4 H; *H*‐3′, C*H_2_*‐5′, C*H*CH_3_), 3.97–3.92 (m, 1 H; *H*‐4′), 2.22 (s, 3 H; COC*H*_3_), 2.12–2.06 (m, 1 H; C*H_2a_*‐2′), 1.40–1.06 (m, 4 H; C*H_2b_*‐2′, CHC*H*_3_), 0.88 (s, 4.5 H; C(C*H*_3_)_3_), 0.87 (s, 4.5 H; C(C*H*_3_)_3_), 0.05 (s, 1.5 H; Si(C*H*_3_)_2_), 0.03 (s, 1.5 H; Si(C*H*_3_)_2_), 0.02 (s, 1.5 H; Si(C*H*_3_)_2_), −0.08 ppm (s, 1.5 H; Si(C*H*_3_)_2_); ^31^P NMR (202 MHz, CD_3_OD): *δ*=4.09 (0.6 P), 3.97 ppm (0.4 P); MS (ES^+^): *m*/*z* (%): 751.85 [*M*+H]^+^ (50), 773 [*M*+Na]^+^ (100).

#### Standard Procedure 3: Synthesis of Phosphoramidates 21 a–f, 25 a and 25 g

*N*‐Acetyl‐3′‐*O*‐silyl‐pseudoisocytidine phosphoramidates **20 a**–**f** or 3′‐*O*‐silyl‐pseudoisocytidine phosphoramidates **24 a** and **24 g** were treated with TFA/CH_2_Cl_2_ (1:1 v/v) at 0 °C. The resulting reaction mixture was stirred at 0 °C for 6 h. After the reaction was completed the solvents were evaporated and the residue was purified by preparative HPLC to afford **21 a**–**f** and **25 a** and **25 g**, respectively.

#### (2*S*)‐Benzyl 2‐[({[(2′*R*,3′*S*,5′*R*)‐5‐(2‐Acetamido‐6‐oxo‐1,6‐dihydropyrimidin‐5‐yl)‐3′‐hydroxytetrahydrofuran‐2′yl]methoxy}‐ (phenoxy)phosphoryl)amino]propanoate (21 a)

Prepared according to standard procedure 3 from compound **20 a** (0.022 g, 0.031 mmol) and TFA/CH_2_Cl_2_ (1:1 v/v, 0.3 mL). After workup, the crude material was purified by preparative HPLC (H_2_O/CH_3_CN from 90:10 to 0:100 in 30 min, flow=20 mL min^−1^, *λ*=280 nm) to yield **21 a** as a white solid (9.2 mg, 50 % yield). ^1^H NMR (500 MHz, CD_3_OD): *δ*=7.88 (s, 1 H; *H*‐6), 7.35–7.29 (m, 7 H; *H*‐Ar), 7.21–7.15 (m, 3 H; *H*‐Ar), 5.17–5.05 (m, 3 H; *H*‐1′, OC*H*_2_Ph), 4.31–4.30 (m, 1 H; *H*‐3′) 4.25–4.11 (m, 2 H; C*H_2_*‐5′), 4.05–4.00 (m, 2 H; *H*‐4′, C*H*CH_3_), 2.36–2.30 (m, 1 H; C*H_2a_*‐2′), 2.21 (s, 1.2 H; COC*H*_3_), 2.20 (s, 1.8 H; COC*H*_3_), 1.81–1.75 (m, 1 H; C*H_2b_*‐2′), 1.37 (d, *J=*7.1 Hz, 1.2 H; CHC*H*_3_), 1.35 ppm (d, *J=*7.2 Hz, 1.2 H; CHC*H*_3_); ^13^C NMR (125 MHz, CD_3_OD): *δ*=175.0 (*C*OCH_3_), 175.0 (*C*OCH_3_), 174.9 (d, *J*_*C–P*_=6.13 Hz; *C*OCH_2_Ph), 167.6 (*C*‐4), 152.2 (d, *J*_*C–P*_=7.2 Hz; *C‐ipso*‐Ph), 152.2 (d, *J*_*C–P*_=7.09 Hz; *C‐ipso*‐Ph), 154.1 (*C*‐6), 152.0 (*C*‐2), 137.2 (*C‐ipso*‐O*C*H_2_Ph), 130.8, 129.6, 129.6, 129.4, 129.3, 129.3, 129.2 (*C*H‐Ar), 124.2 (*C‐5*), 124.1 (*C‐5*), 121.5 (d, *J*_*C–P*_=4.5 Hz; *C*H‐Ph), 121.6 (d, *J*_*C–P*_=4.5 Hz; *C*H‐Ph), 86.3 (d, *J*_*C–P*_=2.3 Hz; *C*‐4′), 86.2 (d, *J*_*C–P*_=2.3 Hz; *C*‐4′), 75.8 (*C*‐1′), 75.8 (*C*‐1′), 74.0 (*C*‐3′), 73.9 (*C*‐3′), 68.1 (d, *J*_*C–P*_=5.6 Hz, *C*‐5′), 68.2 (d, *J*_*C–P*_=5.6 Hz, *C*‐5′), 67.9 (O*C*H_2_Ph), 67.7 (O*C*H_2_Ph), 51.7 (*C*HCH_3_), 51.6 (*C*HCH_3_), 42.8 (*C*‐2′), 42.0 (*C*‐2′), 23.9 (CO*C*H_3_), 20.4 (d, *J*_*C–P*_=7.1 Hz; CH*C*H_3_), 20.4 ppm (d, *J*_*C–P*_=7.4 Hz; CH*C*H_3_); ^31^P NMR (202 MHz, CD_3_OD): *δ*=3.94 (0.7 P), 3.52 ppm (0.3 P); MS (ES^+^): *m*/*z* (%): 587.18 [*M*+H]^+^ (30), 609.53 [*M*+Na]^+^ (100); reversed‐phase HPLC, eluting with H_2_O/CH_3_CN from 90:10 to 0:100 in 30 min, flow=1 mL min^−1^, *λ*=280 nm, *t*_R_=12.79.

#### (2*S*)‐Hexyl 2‐[({[(2′*R*,3′*S*,5′*R*)‐5‐(2‐Acetamido‐6‐oxo‐1,6‐dihydropyrimidin‐5‐yl)‐3′‐hydroxytetrahydrofuran‐2′‐yl]methoxy}(phenoxy)phosphoryl)amino]propanoate (21 b)

Prepared according to standard procedure 3 from compound **20 b** (0.034 g, 0.049 mmol) and TFA/CH_2_Cl_2_ (1:1 v/v, 0.5 mL). After workup, the crude was purified by preparative HPLC (H_2_O/CH_3_CN from 90:10 to 0:100 in 30 min, flow=20 mL min^−1^, *λ*=280 nm) to provide **21 b** as a solid (17 mg, 60 % yield). ^1^H NMR (500 MHz, CD_3_OD): *δ*=7.90 (s, 1 H; *H*‐6), 7.37–7.31 (m, 2 H; *H*‐Ar), 7.24–7.15 (m, 3 H; *H*‐Ar), 5.12 (dd, *J=*9.8, 5.6 Hz, 0.7 H; *H*‐1′), 5.12 (dd, *J=*9.9, 5.9 Hz, 0.3 H; *H*‐1′), 4.34–4.32 (m, 1 H; *H*‐3′) 4.29–4.17 (m, 2 H; C*H*_2_‐5′), 4.13–4.05 (m, 3 H, *H*‐4′; OC*H*_2_), 4.00–3.93 (m, 1 H; C*H*CH_3_), 2.37–2.30 (m, 1 H; C*H_2a_*‐2′), 2.23 (s, 1.2 H; COC*H*_3_), 2.22 (s, 1.8 H; COC*H*_3_), 1.84–1.79 (m, 1 H; C*H_2b_*‐2′) 1.65–1.59 (m, 2 H; OCH_2_C*H*_2_), 1.40–1.28 (m, 9 H; (C*H*_2_)_3_C*H*_3_, CHC*H*_3_), 0.94–0.86 ppm (m, 3 H; CH_2_C*H*_3_); ^13^C NMR (125 MHz, CD_3_OD): *δ*=175.3 (*C*OCH_3_), 175.2 (*C*OCH_3_), 175.0 (*C*O_2_‐hexyl), 175.0 (*C*O_2_‐hexyl), 165.5 (*C*‐4), 152.3 (d, *J*_*C–P*_=6.5 Hz; *C‐ipso*‐Ph), 152.2 (d, *J*_*C–P*_=7.5 Hz; *C‐ipso*‐Ph), 154.2 (*C*‐6), 152.1 (*C*‐2), 130.8, 126.2, 126.1 (*C*H‐Ph), 124.2 (*C‐5*), 124.2 (*C‐5*), 121.5 (d, *J*_*C–P*_=4.6 Hz; *C*H‐Ph), 121.5 (d, *J*_*C–P*_=4.7 Hz; *C*H‐Ph), 86.3 (d, *J*_*C–P*_=2.3 Hz; *C*‐4′), 86.3 (d, *J*_*C–P*_=2.3 Hz; *C*‐4′), 75.9 (*C*‐1′), 75.8 (*C*‐1′), 74.1 (*C*‐3′), 73.9 (*C*‐3′), 68.1 (d, *J*_*C–P*_=5.9 Hz; *C*‐5′), 68.3 (d, *J*_*C–P*_=6.2 Hz; *C*‐5′), 66.5 (O*C*H_2_), 66.5 (O*C*H_2_), 51.7 (*C*HCH_3_), 51.6 (*C*HCH_3_), 41.1 (*C*‐2′), 42.0 (*C*‐2′), 32.6, 29.7, 26.6 (*C*H_2_), 23.9 (CO*C*H_3_), 23.6 (*C*H_2_CH_3_), 20.6 (d, *J*_*C–P*_=6.5 Hz; CH*C*H_3_), 20.5 (d, *J*_*C–P*_=7.3 Hz; CH*C*H_3_), 14.3 ppm (CH_2_ *C*H_3_); ^31^P NMR (202 MHz, CD_3_OD): *δ*=3.88 (0.6 P), 3.58 ppm (0.4 P); MS (ES^+^): *m*/*z* (%): 581.23 [*M*+H]^+^ (34), 603.57 [*M*+Na]^+^ (100); reversed‐phase HPLC, eluting with H_2_O/CH_3_CN from 90:10 to 0:100 in 30 min, flow=1 mL min^−1^, *λ*=254 nm, *t*_R_=15.11.

#### (2*S*)‐Pentyl 2‐[({[(2′*R*,3′*S*,5′*R*)‐5‐(2‐Acetamido‐6‐oxo‐1,6‐dihydropyrimidin‐5‐yl)‐3′‐hydroxytetrahydrofuran‐2′‐yl]methoxy}‐ (phenoxy)phosphoryl)amino]propanoate (21 c)

Prepared according to standard procedure 3 from compound **20 c** (0.050 g, 0.078 mmol) and TFA/CH_2_Cl_2_ (1:1 v/v, 0.5 mL). After workup, the crude was purified by preparative HPLC (H_2_O/CH_3_CN from 90:10 to 0:100 in 30 min, flow=20 mL min^−1^, *λ*=280 nm) to give **21 c** as a solid (6.5 mg, 16 % yield). ^1^H NMR (500 MHz, CD_3_OD): *δ*=7.90 (s, 0.5 H; *H*‐6), 7.85 (s, 0.5 H; *H*‐6), 7.38–7.29 (m, 2 H; *H*‐Ar), 7.24–7.15 (m, 3 H; *H*‐Ar), 5.12 (dd, *J=*9.8, 5.6 Hz, 0.5 H; *H*‐1′), 5.09 (dd, *J=*9.9, 5.9 Hz, 0.5 H; *H*‐1′), 4.35–4.32 (m, 1 H; *H*‐3′) 4.30–4.21 (m, 2 H; C*H*_2_‐5′), 4.15–4.05 (m, 3 H; *H*‐4′, OC*H*_2_), 4.00–3.94 (m, 1 H; C*H*CH_3_), 2.37–2.31 (m, 1 H; C*H_2a_*‐2′), 2.23 (s, 1.5 H; COC*H*_3_), 2.22 (s, 1.5 H; COC*H*_3_), 1.84–1.72 (m, 1 H; C*H_2b_*‐2′), 1.64–1.61 (m, 2 H; OCH_2_C*H*_2_), 1.40–1.28 (m, 9 H; (C*H*_2_)_3_CH_3_, CHC*H*_3_), 0.93–0.91 ppm (m, 3 H; CH_2_C*H*_3_); ^13^C NMR (125 MHz, CD_3_OD): *δ*=176.0 (*C*OCH_3_), 175.2 (*C*OCH_3_), 175.0 (*C*O_2_‐pentyl), 175.0 (*C*O_2_‐pentyl), 165.5 (*C*‐4), 157.7 (*C*‐6), 152.3 (d, *J*_*C–P*_=6.5 Hz; *C‐ipso*‐Ph), 152.2 (d, *J*_*C–P*_=7.5 Hz; *C‐ipso*‐Ph), 152.1 (*C*‐2), 130.6, 126.2, 126.1 (*C*H‐Ph), 124.1 (*C‐5*), 121.5 (d, *J*_*C–P*_=4.6 Hz; *C*H‐Ph), 121.5 (d, *J*_*C–P*_=4.7 Hz; *C*H‐Ph), 86.3 (d, *J*_*C–P*_=2.3 Hz; *C*‐4′), 75.9 (*C*‐1′), 75.8 (*C*‐1′), 74.1 (*C*‐3′), 74.0 (*C*‐3′), 68.3 (d, *J*_*C–P*_=5.9 Hz; *C*‐5′), 68.1 (d, *J*_*C–P*_=6.2 Hz; *C*‐5′), 66.5 (O*C*H_2_), 51.7 (*C*HCH_3_), 51.6 (*C*HCH_3_), 42.0 (*C*‐2′), 29.4, 29.1 (*C*H_2_), 23.9 (CO*C*H_3_), 23.4 (*C*H_2_CH_3_), 20.6 (d, *J*_*C–P*_=6.5 Hz; CH*C*H_3_), 20.5 (d, *J*_*C–P*_=7.3 Hz; CH*C*H_3_), 14.3 ppm (CH_2_ *C*H_3_); ^31^P NMR (202 MHz, CD_3_OD): *δ*=3.92 (0.5 P), 3.60 ppm (0.5P); MS (ES^+^): *m*/*z* (%): 567.20 [*M*+H]^+^ (34), 589.30 [*M*+Na]^+^ (100); reversed‐phase HPLC, eluting with H_2_O/CH_3_CN from 90:10 to 0:100 in 30 min, flow=1 mL min^−1^, *λ*=254 nm, *t*_R_=17.44.

#### (2*S*)‐Isopropyl 2‐[({[(2′*R*,3′*S*,5′*R*)‐5‐(2‐Acetamido‐6‐oxo‐1,6‐dihydropyrimidin‐5‐yl)‐3′‐hydroxytetrahydrofuran‐2′‐yl]methoxy}‐ (naphthalen‐1‐yloxy)phosphoryl)amino]propanoate (21 d)

Prepared according to standard procedure 3 from compound **20 d** (40 mg, 0.057 mmol) and TFA/CH_2_Cl_2_ (1:1 v/v, 0.3 mL). After workup, the crude was purified by preparative HPLC (H_2_O/MeCN from 90:10 to 0:100 in 30 min, flow=20 mL min^−1^, *λ*=280 nm) to yield **21 d** as a white solid (12.6 mg, 37 % yield). ^1^H NMR (500 MHz, CD_3_OD): *δ*=8.10–7.97 (m, 1 H; *H*‐Naph), 7.75–7.65 (m, 2 H; *H*‐Naph, *H*‐6), 7.59–7.47 (m, 1 H; *H*‐Naph), 7.41–7.37 (m, 3 H; *H*‐Naph), 7.32 (d, *J=*8.12 Hz, 0.75 H; *H*‐Naph), 7.30 (d, *J=*8.12 Hz, 0.25 H; *H*‐Naph), 4.93–4.90 (m, 1 H; *H*‐1′), 4.85–4.79 (m, 1 H; OC*H*(CH_3_)_2_), 4.17–4.12 (m, 2 H; C*H*_2_‐5′), 4.11–4.08 (m, 1 H; *H*‐3′), 3.94–3.87 (m, 2 H; C*H*CH_3_ *, H*‐4′), 2.16–2.10 (m, 1 H; C*H_2a_*‐2′), 2.12 (s, 3 H; COC*H*_3_), 2.08–1.99 (m, 1 H; C*H_2b_*‐2′), 1.24 (d, *J=*7.5 Hz, 3 H; CHC*H*_3_), 1.09 (d, *J=*6.4 Hz, 3 H; OCH(C*H*_3_)_2_), 1.08 ppm (d, *J=*6.5 Hz, 3 H, OCH(C*H*_3_)_2_); ^13^C NMR (125 MHz, CD_3_OD): *δ*=173.6 (*C*OCH_3_), 173.4 (*C*=O), 173.1 (*C*=O), 166.2 (*C*‐4), 154.2 (*C*‐6), 152.2 (*C*‐2), 146.7 (d, *J*_C–P_=8.3 Hz; *C*‐*ipso*‐Naph), 134.9 (*C*‐Naph), 127.5 (*C*H‐Naph), 126.5 (d, *J*_*C–P*_=5.2 Hz; *C*‐Naph), 126.0, 126.0, 125.1, 124.6 (*C*H‐Naph), 123.2 (*C*‐5), 121.3 (*C*H‐Naph), 114.9 (d, *J*_*C–P*_=3.6 Hz; *C*H‐Naph), 85.0 (*C*‐4′), 74.4 (*C*‐1′), 72.6 (*C*‐3′), 68.8 (O*C*H), 66.9 (d, *J*_*C–P*_=5.02 Hz; *C*‐5′), 50.6 (*C*HCH_3_), 40.2 (*C*‐2′), 22.5 (CO*C*H_3_), 20.5 (OCH(*C*H_3_)_2_), 20.4 (OCH(*C*H_3_)_2_), 19.1 (d, *J*_*C–P*_=7.3 Hz; CH*C*H_3_), 19.0 ppm (d, *J*_*C–P*_=7.3 Hz; CH*C*H_3_); ^31^P NMR (202 MHz, CD_3_OD): *δ*=4.34 ppm; MS (ES^+^): *m*/*z* (%): 611.20 [*M*+Na]^+^ (100); reversed‐phase HPLC, eluting with H_2_O/CH_3_CN from 90:10 to 0:100 in 30 min, flow=1 mL min^−1^, *λ*=254 nm, *t*_R_=18.72 min.

#### (2*S*)‐Neopentyl 2‐[({[(2′*R*,3′*S*,5′*R*)‐5‐(2‐Acetamido‐6‐oxo‐1,6‐dihydropyrimidin‐5‐yl)‐3′‐hydroxytetrahydrofuran‐2′‐yl]me‐ thoxy}(naphthalen‐1‐yloxy)phosphoryl)amino]propanoate (21 e)

Prepared according to standard procedure 3 from compound **20 e** (42 mg, 0.056 mmol) and TFA/CH_2_Cl_2_ (1:1 v/v, 0.3 mL). After workup, the crude was purified by preparative HPLC (H_2_O/CH_3_CN from 90:10 to 0:100 in 30 min, flow=20 mL min^−1^, *λ*=280 nm) to give **21 e** as a solid (16.0 mg, 46 % yield). ^1^H NMR (500 MHz, CD_3_OD): *δ*=8.02–7.99 (m, 1 H; *H*‐Naph), 7.75–7.70 (m, 1 H; *H*‐Naph), 7.66 (s, 1 H; *H*‐6), 7.57 (d, *J=*7.8 Hz, 0.7 H; *H*‐Naph), 7.52 (d, *J=*8.4 Hz, 0.3 H; *H*‐Naph), 7.40–7.34 (m, 3 H; *H*‐Naph), 7.32–7.28 (m, 0.7 H; *H*‐Naph), 7.31–7.28 (m, 0.3 H; *H*‐Naph), 4.89 (dd, *J=*10.1, 6.2 Hz, 1 H; *H*‐1′), 4.20–4.12 (m, 2 H; C*H_2_*‐5′), 4.13–4.07 (m, 1 H; *H*‐3′), 4.00–3.96 (m, 1 H; C*H*CH_3_), 3.92–3.90 (m, 1 H; *H*‐4′), 3.73, 3.66 (AB system*, J=*10.5 Hz, 1 H; OC*H*_2_C(CH_3_)_3_), 3.72, 3.62 (AB system*, J=*10.5 Hz, 1 H; OC*H*_2_C(CH_3_)_3_), 2.16–2.10 (m, 1 H; C*H_2a_*‐2′), 2.10 (s, 2 H; COC*H*_3_), 2.09 (s, 1 H; COC*H*_3_), 2.16–2.10 (m, 1 H; C*H_2b_*‐2′), 1.26 (d, *J=*7.18 Hz, 1.2 H; CHC*H*_3_), 1.24 (d, *J=*7.3 Hz, 1.8 H; CHC*H*_3_), 0.79 (s, 3 H; OCH_2_C(C*H*_3_)_3_), 0.81 ppm (s, 6 H; OCH_2_C(C*H*_3_)_3_); ^13^C NMR (125 MHz, CD_3_OD): *δ*=173.7 (*C*OCH_3_), 171.2 (*C*OCH_2_C(CH_3_)_3_), 170.9 (*C*OCH_2_C(CH_3_)_3_), 159.2 (*C*‐4), 153.5 (*C*‐6), 152.1 (*C*‐2), 146.7 (d, *J*_*C–P*_=6.8 Hz; *C*‐*ipso*‐Naph), 134.9 (*C*‐Naph), 127.4 (*C*H‐Naph), 126.5 (d, *J*_*C–P*_=5.2 Hz, *C*‐Naph), 126.3, 126.1, 125.1, 124.6 (*C*H‐Naph), 123.1 (*C*‐5), 115.0 (d, *J*_*C–P*_=3.6 Hz; *C*H‐Naph), 85.0 (*C*‐4′), 74.4 (*C*‐1′), 74.4 (*C*‐1′), 74.4 (O*C*H_2_C(CH_3_)_3_), 72.6 (*C*‐3′), 66.9 (d, *J*_*C–P*_=5.0 Hz; *C*‐5′), 66.9 (d, *J*_*C–P*_=5.0 Hz; *C*‐5′), 50.5 (*C*HCH_3_), 50.5 (*C*HCH_3_), 40.8 (*C*‐2′), 30.9 (OCH_2_ *C*(CH_3_)_3_), 25.3 (OCH_2_C(*C*H_3_)_3_), 22.5 (CO*C*H_3_), 19.2 ppm (d, *J*_*C–P*_=7.3 Hz; CH*C*H_3_). ^31^P NMR (202 MHz, CD_3_OD): *δ*=4.36 (0.7 P), 4.09 ppm (0.3 P); MS (ES^+^): *m*/*z* (%): 639.21 [*M*+Na]^+^ (100); reversed‐phase HPLC, eluting with H_2_O/CH_3_CN from 90:10 to 0:100 in 30 min, flow=1 mL min^−1^, *λ*=254 nm, *t*_R_=11.95 min.

#### (2*S*)‐Benzyl 2‐[({[(2′*R*,3′*S*,5′*R*)‐5‐(2‐Acetamido‐6‐oxo‐1,6‐dihydropyrimidin‐5‐yl)‐3′‐hydroxytetrahydrofuran‐2′‐yl]methoxy}‐ (naphthalen‐1‐yloxy)phosphoryl)amino]propanoate (21 f)

Prepared according to standard procedure 3 from the compound **20 f** (98 mg, 0.130 mmol) and and TFA/CH_2_Cl_2_ (1:1 v/v, 0.3 mL). After workup, the crude material was purified by preparative HPLC (H_2_O/CH_3_CN from 90:10 to 0:100 in 30 min, flow=20 mL min^−1^, *λ*=280 nm) to give **21 f** as a white solid (20.1 mg, 25 % yield). ^1^H NMR (500 MHz, CD_3_OD): *δ*=8.13–8.11 (m, 1 H; *H*‐Naph), 7.86–7.84 (m, 2 H; *H*‐Naph, *H*‐6), 7.71–7.64 (m, 1 H; *H*‐Naph), 7.51–7.46 (m, 3 H; *H*‐Naph), 7.32 (d, *J=*8.12 Hz, 0.65 H; *H*‐Naph), 7.30 (d, *J=*8.12 Hz, 0.35 H; *H*‐Naph), 7.33–7.28 (m, 5 H; *H*‐Ph), 5.14–5.07 (m, 2 H; OC*H*_2_Ph), 5.03 (dd, *J=*8.6, 5.5 Hz, 1 H; *H*‐1′), 4.25–4.20 (m, 3 H; C*H_2_*‐5′, *H*‐3′), 4.15–4.09 (m, 1 H; C*H*CH_3_), 4.03–4.00 (m, 1 H; *H*‐4′), 2.21 (s, 3 H; COC*H*_3_), 2.19–2.15 (m, 1 H; C*H_2a_*‐2′), 1.49–1.36 (m, 1 H; C*H_2b_*‐2′), 1.37 (d, *J=*7.5 Hz, 3 H; CHC*H*_3_); ^13^C NMR (125 MHz, CD_3_OD): *δ*=173.6 (*C*OCH_3_), 173.6 (*C*=O), 173.5 (*C*=O), 166.2 (*C*‐4), 154.2 (*C*‐6), 150.6 (*C*‐2), 146.7 (d, *J*_*C–P*_=8.3 Hz; *C*‐*ipso*‐Naph), 146.6 (d, *J*_*C–P*_=8.3 Hz; *C‐ipso*‐Naph), 135.8, 134.9, 134.8 (*C*‐Naph), 128.2 128.1, 127.9, 127.9, 127.8, 127.4 (*C*H‐Naph), 126.5 (d, *J*_*C–P*_=5.17 Hz; *C*‐Naph), 126.4, 126.3, 126.1, 125.1 (*C*H‐Naph), 124.6 (*C*‐5), 124.5 (*C*‐5), 121.3, 121.3 (*C*H‐Naph), 115.1 (d, *J*_*C–P*_=3.6 Hz; *C*H‐Naph), 115.0 (d, *J*_*C–P*_=3.6 Hz; *C*H‐Naph), 85.1 (*C*‐4′), 85.0 (*C*‐4′), 74.3 (*C*‐1′), 74.2 (*C*‐1′), 72.7 (*C*‐3′), 72.6 (*C*‐3′), 69.0 (d, *J*_*C–P*_=5.0 Hz; *C*‐5′), 66.9 (d, *J*_*C–P*_=5.0 Hz, *C*‐5′), 66.6 (O*C*H_2_Ph), 66.6 (O*C*H_2_Ph), 50.5 (*C*HCH_3_), 50.4 (*C*HCH_3_), 40.7 (*C*‐2′), 40.5 (*C*‐2′), 22.5 (CO*C*H_3_), 19.0 (d, *J*_*C–P*_=7.3 Hz; CH*C*H_3_), 18.9 ppm (d, *J*_*C–P*_=7.3 Hz; CH*C*H_3_); ^31^P NMR (202 MHz, CD_3_OD): *δ*=4.33 (0.8 P), 3.98 ppm (0.2 P); MS (ES^+^): *m*/*z* (%): 659.20 [*M*+Na]^+^ (100); reversed‐phase HPLC, eluting with H_2_O/CH_3_CN from 90:10 to 0:100 in 30 min, flow=1 mL min^−1^, *λ*=254 nm, *t*_R_=17.45 min.

#### 5 ‐Amino‐5‐((2′*R*,4′*S*,5′*R*)‐4‐[(*tert*‐butyldimethylsilyl)oxy]‐5′‐{[(*tert*‐butyldimethylsilyl)oxy]methyl}tetrahydrofuran‐2′‐yl)pyrimidin‐4(3*H*)‐one (22)

Ammonia (7 m) in MeOH (1 mL) was added to a solution of 2‐*N*‐acetyl‐2′‐deoxy‐3′,5′‐*O*‐bis(*tert*‐butyldimethylsilyl)pseudoisocytidine (**16**, 0.55 g, 1.1 mmol) in CH_3_OH (5 mL) at 0 °C under an argon atmosphere. After 30 min the reaction mixture was allowed to warm to room temperature and was stirred for 12 h. The volatiles were removed under vacuum and the crude material was purified by flash column chromatography using CH_2_Cl_2_/CH_3_OH (95:5) as the eluent to give **22** as an oil (0.455 g, 90 % yield). ^1^H NMR (500 MHz, CD_3_OD): *δ*=7.54 (s, 1 H; *H*‐6), 4.94 (dd, *J=*10.2, 6.0 Hz, 1 H; *H*‐1′), 4.31–4.28 (m, 1 H; *H*‐3′), 3.74–3. 68 (m, 1 H; *H*‐4′), 3.61 (dd, *J=*11.0, 4.0 Hz, 1 H; C*H_2a_*‐5′), 3.54 (dd, *J=*11.0, 5.5 Hz, 1 H; C*H_2b_*‐5′), 2.09 (dd, *J=*12.5, 6.0 Hz, 1 H; C*H_2a_*‐2′), 1.82–1.76 (m, 1 H; C*H_2b_*‐2′), 0.81 (s, 9 H; SiC(C*H*_3_)_3_), 0.80 (s, 9 H; SiC(C*H*_3_)_3_), 0.02 (s, 3 H; Si(C*H*_3_)_2_), 0.00 (s, 3 H; Si(C*H*_3_)_2_), −0.01 (s, 3 H; Si(C*H*_3_)_2_), −0.02 ppm (s, 3 H; Si(C*H*_3_)_2_); ^13^C NMR (125 MHz, CD_3_OD): *δ*=165.3 (*C*‐4), 155.9 (*C*‐2), 128.7 (*C*‐6), 128.7 (*C*‐6), 116.0 (*C*‐5), 87.5 (*C*‐4′), 74.2 (*C*‐1′), 74.1 (*C*‐3′), 63.4 (*C*‐5′), 41.0 (*C*‐2′), 25.1 (SiC(*C*H_3_)_3_), 25.0 (SiC(*C*H_3_)_3_), 17.8 (Si*C*(CH_3_)_3_), 17.5 (Si*C*(CH_3_)_3_), −5.7 (Si(*C*H_3_)_2_), −5.9 (Si*C*H_3_)_2_), −6.6 (Si*C*H_3_)_2_), −6.6 ppm (Si*C*H_3_)_2_); MS (ES^+^): *m*/*z* (%): 456.3 [*M*+H]^+^ (100); reversed‐phase HPLC, eluting with H_2_O/CH_3_CN from 90:10 to 0:100 in 30 min, flow=1 mL min^−1^, *λ*=254 nm, *t*_R_=19.19 min.

#### 6 ‐Amino‐5‐{(2′*R*,4′*S*,5′*R*)‐4′‐[(*tert*‐butyldimethylsilyl)oxy]‐5′‐(hydroxymethyl)tetrahydrofuran‐2′‐yl}pyrimidin‐4(3*H*)‐one (23)

A mixture of TFA and H_2_O (1:1 v/v, 2.0 mL) was added dropwise to a solution of **22** (0.244 g, 0.535 mmol) in THF (4 mL) at 0 °C. The reaction was stirred at room temperature for 2 h under an argon atmosphere. The reaction mixture was quenched with NaHCO_3_ and concentrated under reduced pressure to afford **23** as glassy solid, which was used in the next step without further purification (0.123 g, 67 %). ^1^H NMR (500 MHz, CD_3_OD): *δ*=7.61 (s, 1 H; *H*‐6), 4.92 (dd, *J=*10.2, 6.0 Hz, 1 H; *H*‐1′), 4.35–4.31 (m, 1 H; *H*‐3′), 3.82‐ 3.74 (m, 1 H; *H*‐4′), 3.58 (dd, *J=*11.5, 4.5 Hz, 1 H; C*H_2a_*‐5′), 3.54 (dd, *J=*11.5, 4.5 Hz, 1 H; C*H_2b_*‐5′), 2.16–2.10 (m, 1 H; C*H_2a_*‐2′), 1.88–1.82 (m, 1 H; C*H_2b_*‐25′), 0.85 (s, 9 H; SiC(C*H*_3_)_3_), 0.06 (s, 3 H; Si(C*H*_3_)_2_), 0.01 ppm (s, 3 H; Si(C*H*_3_)_2_); ^13^C NMR (125 MHz, CD_3_OD): *δ*=161.3 (*C*‐4), 150.3 (*C*‐6), 149.8 (*C*‐2), 123.1 (*C*‐5), 88.5 (*C*‐4′), 85.7 (*C*‐1′), 72.2 (*C*‐3′), 70.0 (*C*‐5′), 37.4 (*C*‐2′), 24.0 (SiC(*C*H_3_)_3_), 19.7 (Si*C*(CH_3_)_3_), −6.1 (Si(*C*H_3_)_2_), −6.4 ppm (Si(*C*H_3_)_2_); reversed‐phase HPLC, eluting with H_2_O/CH_3_CN from 90:10 to 0:100 in 30 min, flow=1 mL min^−1^, *λ*=254 nm, *t*_R_=10.8 min.

#### (2*S*)‐Benzyl 2‐{[({(2′*R*,3′*S*,5′*R*)‐5‐(2‐Amino‐6‐oxo‐1,6‐dihydropyrimidin‐5‐yl)‐3′‐[(*tert*‐butyldimethylsilyl)oxy]tetrahydrofuran‐2′‐yl}methoxy)(phenoxy)phosphoryl]amino}propanoate (24 a)

Prepared according to standard procedure 2 from nucleoside **23** (0.035 g, 0.1 mmol), **18 a** (0.071 g, 0.2 mmol) in anhydrous THF (2 mL) and *t*BuMgCl in THF (1 m, 0.13 mL). After workup, the crude was purified by column chromatography on silica gel using CH_2_Cl_2_/CH_3_OH (92:8) as the eluent to give **24 a** as a solid (0.0223 g, 41 %). ^1^H NMR (500 MHz, CD_3_OD): *δ*=7.56 (s, 1 H; *H*‐6), 7.26–7.19 (m, 7 H; *H*‐Ph), 7.12–7.06 (m, 3 H; *H*‐Ph), 5.07–5.01 (m, 2 H; OC*H*_2_Ph), 4.94 (dd, *J=*10.0, 6.0 Hz, 1 H; *H*‐1′), 4.29–4.21 (m, 1 H; *H*‐3′), 4.05–4.01 (m, 2 H; C*H*_2_‐5′), 3.96–388 (m, 1 H; C*H*CH_3_), 3.86–3.83 (m, 1 H; *H*‐4′), 2.10–2.06 (m, 0.3 H; *H*‐2′a), 2.04–2.01 (m, 0.7 H; *H*‐2′a), 1.74–1.79 (m, 1 H; *H*‐2′b), 1.29–1.25 (m, 3 H; CHC*H*_3_), 0.84 (s, 2.7 H; SiC(C*H*_3_)_3_), 0.82 (s, 6.3 H; SiC(C*H*_3_)_3_), 0.01 (s, 1.8 H; Si(C*H*_3_)_2_), 0.00 ppm (s, 4.2 H; Si(C*H*_3_)_2_); ^31^P NMR (202 MHz, CD_3_OD): *δ*=3.79 (0.7 P), 3.49 ppm (0.3 P).

#### (2*S*)‐Isopropyl 2‐{[({(2′*R*,3′*S*,5′*R*)‐5‐(2‐Amino‐6‐oxo‐1,6‐dihydropyrimidin‐5‐yl)‐3′‐[(*tert*‐butyldimethylsilyl)oxy]tetrahydrofuran‐2′‐yl}methoxy)(phenoxy)phosphoryl]amino}propanoate (24 g)

Prepared according to standard procedure 2 from nucleoside **23** (0.035 g, 0.1 mmol), **18 g** (0.067 g, 0.2 mmol) in anhydrous THF (1.5 mL) and *t*BuMgCl in THF (1 m, 0.13 mL). After workup, the crude material was purified by column chromatography on silica gel using CH_2_Cl_2_/CH_3_OH (92:8) as an eluent to give **24 g** as a solid (0.020 g, 32 %). ^1^H NMR (500 MHz, CD_3_OD): *δ*=7.56 (br s, 1 H; *H*‐6), 7.26–7.22 (m, 2 H; *H*‐Ph), 7.14–7.07 (m, 3 H; *H*‐Ph), 4.94 (dd, *J=*10.0, 6.0 Hz, 1 H; *H*‐1′), 4.87–4.79 (m, 1 H; OC*H*(CH_3_)_2_), 4.38–4.31 (m, 1 H; *H*‐3′), 4.12–4.03 (m, 2 H; C*H_2_*‐5′), 3.89–3.88 (m, 1 H; *H*‐4′), 3.83–3.77 (m, 1 H; C*H*CH_3_), 2.13–2.00 (m, 1 H; C*H_2a_*‐2′), 1.79–1.69 (m, 1 H; C*H_2a_*‐2′), 1.25 (d, *J=*7.0 Hz, 0.7 H; CHC*H*_3_), 1.22 (d, *J=*7.0 Hz, 2.3 H; CHC*H*_3_), 1.14 (d, *J=*6.0 Hz, 3 H; OCH(C*H*_3_)_2_), 1.12 (d, *J=*6.0 Hz, 3 H; OCH(C*H*_3_)_2_), 0.82 (s, 2.3 H; SiC(C*H*_3_)_3_), 0.81 (s, 6.7 H; SiC(C*H*_3_)_3_), 0.02 (s, 0.7 H; Si(C*H*_3_)_2_), 0.05 (s, 0.7 H; Si(C*H*_3_)_2_), −0.03 (s, 2.3 H; Si(C*H*_3_)_2_), −0.07 ppm (s, 2.3 H; Si(C*H*_3_)_2_); ^31^P NMR (202 MHz, CD_3_OD): *δ*=3.87 (0.3 P), 3.57 ppm (0.7 P); reversed‐phase HPLC, eluting with H_2_O/CH_3_CN from 90:10 to 0:100 in 30 min, flow=1 mL min^−1^, *λ*=254 nm, *t*_R_=13.42 min.

#### (2*S*)‐Benzyl 2‐[({[(2′*R*,3′*S*,5′*R*)‐5‐(2‐Amino‐6‐oxo‐1,6‐dihydropyrimidin‐5‐yl)‐3′‐hydroxytetrahydrofuran‐2′‐yl]methoxy}(phenoxy)phosphoryl)amino]propanoate (25 a)

Trimethylsilyl trifluoromethanesulfonate (14.5 μL, 0.08 mmol) was added dropwise to a solution of **24 a** (22.3 mg, 0.034 mmol) in CH_2_Cl_2_ (1.5 mL) at −78 °C. The reaction was stirred for 2 h under an argon atmosphere. The solvent was removed under reduced pressure and the crude material was purified by preparative HPLC to yield **25 a** as a white solid (11.3 mg, 62 %). ^1^H NMR (500 MHz, CD_3_OD): *δ*=7.52 (s, 0.9 H; *H*‐6), 7.45 (s, 0.1 H; *H*‐6), 7.25–7.18 (m, 7 H; *H*‐Ph), 7.09–7.05 (m, 3 H; *H*‐Ph), 5.05–4.99 (m, 2 H; OC*H*_2_Ph), 4.91 (dd, *J=*10.1, 6.1 Hz, 1 H; *H*‐1′), 4.22–4.20 (m, 1 H; *H*‐3′), 4.13–4.02 (m, 2 H; C*H_2_*‐5′), 3.97–3.95 (m, 1 H; *H*‐4′), 3.91–3.85 (m, 1 H; C*H*CH_3_), 2.20 (ddd, *J=*13.0, 5.5, 2.0 Hz, 1 H; C*H_2_*‐2′a), 1.69 (ddd, *J=*13.0, 10.0, 6.0 Hz, 1 H; C*H_2_*‐2′b), 1.28–1.21 ppm (m, 3 H; CHC*H*_3_); ^13^C NMR (125 MHz, CD_3_OD): *δ*=170.4 (*C*OCH_2_Ph), 169.9 (*C*OCH_2_Ph), 159.7 (*C*‐4), 155.4 (*C*‐6), 152.5 (*C*‐2), 152.1 (d, *J*_*C–P*_=7.1 Hz; *C‐ipso*‐Ph), 137.1 (*C‐ipso*‐OCH_2_Ph), 129.5, 129.4, 128.2, 128.0, 127.9, 127.7, 127.6, 125.6 (C*H*‐Ar), 124.8 (*C*‐5), 120.1 (d, *J*_*C–P*_=4.3 Hz; *C*H‐Ph), 120.1 (d, *J*_*C–P*_=4.5 Hz; *C*H‐Ph), 85.2 (*C*‐4′), 73.7 (*C*‐1′), 72.6 (*C*‐3′), 72.1 (*C*‐3′), 67.0 (O*C*H_2_Ph), 66.6 (d, *J*_*C–P*_=5.7 Hz, *C*‐5′), 50.5 (*C*HCH_3_), 40.6 (*C*‐2′), 18.9 (d, *J=*5.9 Hz, CH*CH*_3_), 18.9 ppm (d, *J=*5.87 Hz, CH*C*H_3_); ^31^P NMR (202 MHz, CD_3_OD): *δ*=3.93 ppm (1P); MS (ES^+^): *m*/*z* (%): 545.2 [*M*+H]^+^ (100); reversed‐phase HPLC, eluting with H_2_O/CH_3_CN from 90:10 to 0:100 in 30 min, flow=1 mL min^−1^, *λ*=254 nm, *t*_R_=9.51 min.

#### (2*S*)‐Isopropyl 2‐[({[(2′*R*,3′*S*,5′*R*)‐5‐(2‐Amino‐6‐oxo‐1,6‐dihydropyrimidin‐5‐yl)‐3′‐hydroxytetrahydrofuran‐2′‐yl]methoxy}(phenoxy)phosphoryl)amino]propanoate (25 g)

Prepared according to standard procedure 3 from compound **24 g** (10 mg, 0.016 mmol) and TFA/CH_2_Cl_2_ (1:1 v/v, 0.6 mL). After workup, the crude material was purified by preparative HPLC to give **25 g** as a solid (4.0 mg, 50 %). ^1^H NMR (500 MHz, CD_3_OD): *δ*=7.68 (s, 0.8 H; *H*‐6), 7.67 (s, 0.2 H; *H*‐6), 7.26–7.20 (m, 2 H; *H*‐Ph), 7.15–7.02 (m, 3 H; *H*‐Ph), 5.07 (dd, *J=*10.0, 5.5 Hz, 1 H; *H*‐1′), 4.98–4.96 (m, 1 H; OC*H*(CH_3_)_2_), 4.33–4.28 (m, 1 H; *H*‐3′), 4.23–4.19 (m, 2 H; C*H*_2_‐5′), 4.05–4.01 (m, 1 H; *H*‐4′), 3.92–3.87 (m, 1 H; C*H*CH_3_), 2.32–2.20 (m, 2 H; C*H*_2_‐2′), 1.90–1.80 (m, 2 H; C*H*_2_‐5′), 1.37 (d, *J=*7.0 Hz, 0.6 H; CHC*H*_3_), 1.34 (d, *J=*7.0 Hz, 2.4 H; CHC*H*_3_), 1.25 (d, *J=*6.5 Hz, 3 H; OCH(C*H*_3_)_2_), 1.23 ppm (d, *J=*6.5 Hz, 3 H; CH(C*H*_3_)_2_); ^13^C NMR (125 MHz, CD_3_OD): δ=170.2 (*C*OCH(CH_3_)_2_), 159.2 (*C*‐4), 153.5 (*C*‐6), 152.1 (*C*‐2), 141.1 (d, *J*_*C‐P*_=4.5 Hz; *C*‐*ipso*‐Ph), 129.4, 126.8 (*CH*‐Ph), 123.5 (*C*‐5), 120.2 (d, *J*_*C–P*_=5.7 Hz; *C*H‐Ph), 120.1 (d, *J*_*C–P*_=4.5 Hz; *CH*‐Ph), 84.8 (d, *J*_*C–P*_=8.4 Hz; *C*‐4′), 84.8 (d, *J*_*C–P*_=8.4 Hz; *C*‐4′), 74.7 (*C*‐1′), 72.8 (*C‐3′*), 68.8 (O*CH*(CH_3_)_2_), 66.8 (d, *J*_*C–P*_=5.7 Hz; *C*‐5′), 50.5 (*C*HCH_3_), 40.5 (*C*‐2′), 39.5 (*C*‐2′), 20.6 (OCH(*C*H_3_)_2_), 20.5 (OCH(*C*H_3_)_2_), 19.5 (d, *J*_*C–P*_=7.5 Hz; CH*C*H_3_), 19.0 ppm (d, *J*_*C–P*_=7.5 Hz; CH*C*H_3_); ^31^P NMR (202 MHz, CD_3_OD): *δ*=3.91 (0.8 P), 3.69 ppm (0.2 P); MS (ES^−^): *m*/*z* (%): 495.15 [*M*−H]^+^ (100); reversed‐phase HPLC, eluting with H_2_O/CH_3_CN from 90:10 to 0:100 in 30 min, flow=1 mL min^−1^, *λ*=254 nm, *t*_R_=7.75 min.

### Carboxypeptidase Y assay

Trizma buffer (300 μL, pH 7.6) was added to compound **21 e** (5 mg) dissolved in [D_6_]acetone (150 μL). The ^31^P NMR spectrum (202 MHz, 64–128 scans) was recorded at this stage as a reference (blank, *t*=0). To this mixture, a stock solution of carboxypeptidase Y (Sigma–Aldrich, >50 units mg^−1^, dissolved in pH 7.6 Trizma buffer, to a concentration of 50 units mL^−1^, EC 3.4.16.1, 130 μL) was added. ^31^P NMR spectra (128 scans) were recorded with a 3 min delay between experiments for 14 h at 25 °C.

## Conflict of interest

*The authors declare no conflict of interest*.
